# The Adaptation and Tolerance of Major Cereals and Legumes to Important Abiotic Stresses

**DOI:** 10.3390/ijms222312970

**Published:** 2021-11-30

**Authors:** Jagadish Rane, Ajay Kumar Singh, Mahesh Kumar, Karnar M. Boraiah, Kamlesh K. Meena, Aliza Pradhan, P. V. Vara Prasad

**Affiliations:** 1 National Institute of Abiotic Stress Management, Baramati 413115, India; ajay.singh4@icar.gov.in (A.K.S.); mahesagrawal@gmail.com (M.K.); Boraiah.M@icar.gov.in (K.M.B.); kkmeenamicro@gmail.com (K.K.M.); aliza.pradhan@icar.gov.in (A.P.); 2 Department of Agronomy, Kansas State University, Manhattan, KS 66506, USA; vara@ksu.edu

**Keywords:** abiotic stress, drought, salinity, heat, tolerance mechanism, management

## Abstract

Abiotic stresses, including drought, extreme temperatures, salinity, and waterlogging, are the major constraints in crop production. These abiotic stresses are likely to be amplified by climate change with varying temporal and spatial dimensions across the globe. The knowledge about the effects of abiotic stressors on major cereal and legume crops is essential for effective management in unfavorable agro-ecologies. These crops are critical components of cropping systems and the daily diets of millions across the globe. Major cereals like rice, wheat, and maize are highly vulnerable to abiotic stresses, while many grain legumes are grown in abiotic stress-prone areas. Despite extensive investigations, abiotic stress tolerance in crop plants is not fully understood. Current insights into the abiotic stress responses of plants have shown the potential to improve crop tolerance to abiotic stresses. Studies aimed at stress tolerance mechanisms have resulted in the elucidation of traits associated with tolerance in plants, in addition to the molecular control of stress-responsive genes. Some of these studies have paved the way for new opportunities to address the molecular basis of stress responses in plants and identify novel traits and associated genes for the genetic improvement of crop plants. The present review examines the responses of crops under abiotic stresses in terms of changes in morphology, physiology, and biochemistry, focusing on major cereals and legume crops. It also explores emerging opportunities to accelerate our efforts to identify desired traits and genes associated with stress tolerance.

## 1. Introduction

Major abiotic stresses that are likely to be amplified by climate change and can destabilize crop yields include drought, extreme temperatures, flooding, waterlogging, soil salinity, acidity, mineral toxicity, and nutrient deficiency. Global climate change and environmental degradation are intensifying the severity of abiotic stresses that adversely affect the growth, development, and productivity of crop plants. The increasing frequency of extreme weather events due to climate change is expected to cause severe risks to sustainable crop production for major cereal and leguminous crop species. Losses in production caused by abiotic stresses may exceed 40% [1], and hence these stresses could be a constant threat to global food security if not properly managed. The Food and Agricultural Organization (FAO) has emphasized that about a 60% enhancement in food production is needed by 2050 to feed a population of about 9.3 billion. This must be achieved with no adverse effects on the environment, which is threatened by the continuous exploitation of natural resources and the loss of biodiversity. In order to ensure sustainability even under the changing climate, it is essential to develop resilient cultivars of crops and suitable crop production practices with insights on stress tolerance mechanisms and the associated traits. The climate resilience of agriculture depends on crop tolerance to multiple abiotic stresses that cause considerable yield losses individually, as well as in combination. Abiotic stresses negatively impact plant growth, development, reproduction, and ultimately crop productivity. Crops can tolerate certain environmental stresses through their innate adaptation mechanisms, which are driven by various physiological and metabolic processes at the cellular level and are manifested in the whole plant. However, the degree of tolerance and adaptability to abiotic stresses may differ across species and cultivars of crops [2]. It is necessary to understand the adaptive mechanisms of plants to identify traits associated with tolerance to abiotic stresses. Several proteins and genes involved in abiotic stress adaptation and mitigation have been integrated to develop varieties that are tolerant to abiotic stresses [3,4,5,6]. The genetic engineering of crop plants with stress-responsive genes has been demonstrated to enhance the adaptation to various abiotic stresses [1,7,8,9]. Much of the gene identification efforts can be traced to model plants like *Arabidopsis*, and this basic knowledge needs to be accelerated to make the crop plants resilient to abiotic stresses in target growth environments. There is a need to explore those aspects of stress adaptation that have not been effectively utilized or employed to improve key food grain crops.

Microbes that are associated with plants in natural habitats under diverse environments exhibit tremendous capabilities to cope with stresses caused by environmental factors. Since plants must interact with microbes, microbes are believed to modulate plant defense mechanisms to protect against adverse external conditions [10,11]. With better insights into plants’ responses to environmental challenges such as drought, heat, and salinity, improved management practices and tools can be applied to increase stable yields. This review aims to update current knowledge about the impacts of key abiotic stresses in major cereal and legume crops, the mechanisms of tolerance to various abiotic stresses, and the opportunities to translate the knowledge for the development of climate-resilient crop varieties and management practices.

## 2. Abiotic Stresses and Their Impacts on Grain Crops

Plants experience abiotic stresses when they are exposed to supra- or sub-optimal levels of environmental factors such as temperature, soil moisture, and salts in the soil [12,13]. It is increasingly realized that climate change will lead to a decline in crop productivity, mainly by enhancing the frequency and intensity of abiotic stresses such as extreme temperatures, droughts, salinity, and waterlogging. The ability of crops to cope with these challenging situations is the crucial aspect of abiotic stress resilience and stable crop productivity. Hence, genetic improvement has long been a target for crop scientists to make crops more resilient to stresses. There is a need to accelerate the current efforts to develop stress-tolerant genotypes, with a focus on the traits that contribute to the abiotic stress tolerance and grain yield, which is conventionally preferred. This process would be driven by our knowledge about plant mechanisms to survive and grow under the constant changes and extreme environmental conditions at the whole plant, organ, tissue, and cellular levels (Figure S1) [3,14,15,16,17,18,19,20].

### 2.1. Drought

Droughts are a major challenge faced by most food crops that are sensitive to a soil moisture deficit. The impact of droughts on the final yield and various physiological and biochemical processes of crops depends on its intensity, timing, and duration (Table 1 and Table 2). However, its impact is lessened in the cases of adopted and evolved crops under the harsh conditions of semi-arid and arid regions [21]. Both the vegetative phase, as evident from leaf growth, and the reproductive phase, as evident from floral development, is severely affected due to soil moisture deficits [22,23]. The timing and duration of water stress determines the impacts on developmental processes, as evident from changes in the duration of flowering if stress is applied at the early stages of growth, and a reduction in the grain filling duration if the stress occurs at early or terminal reproductive growth stages [23,24]. Extreme drought conditions impair crop morphology, physiology, and duration, while the moisture content plays a vital role in germination as it affects the enzyme activation that determines plant sensitivity during germination. The occurrence of droughts when the grain filling rate is at its peak can accelerate the leaf senescence and result in smaller grains [23,25]. There is a considerable tolerance range to drought stress across the cereals and legume crops and their cultivars [26,27].

### 2.2. High Temperatures

Projected increases in higher ambient temperatures worldwide are likely to drastically reduce crop productivity [1,23,57]. The rise from the seasonal average temperature by 1 °C was shown to reduce cereals’ grain yields by 4.1% to 10.0% [58]. High temperatures can lead to a shorter crop life cycle and, hence, a reduction in cereals’ productivity [23,59,60,61]. In wheat, reductions in grain yield [23,62,63,64] and quality [65] have been reported. This is mainly due to accelerated development [66], reduced photosynthesis [67], and the direct impacts on reproductive processes [23,59]. Losses in grain yields due to high temperature stress on selected cereal and legume crops are shown in Table 3. The tolerance to high temperatures in rice is relatively higher at the early growth stage; however, the crop is highly vulnerable to elevated temperatures at later stages, particularly at flowering [59,68,69]. The high sensitivity of the reproductive stage has been reported even in recently released cultivars of wheat [70]. Sensitive stages and temperature thresholds of key cereals crops such as wheat [71], sorghum [23,72], and finger millet [73] are well quantified. Similarly, the impact of high temperature stress on various physiological, growth, reproductive fertility, and yield components are well documented for major cereals such as rice [68,74], wheat [71,75,76], sorghum [77,78], and pearl millet [79]. Similarly, the impacts on major legume crops are also well documented for crops such as the chickpea [80,81], black gram [82], green gram [83], kidney bean [84], soybean [85], peanut [86,87,88,89], and lentil [90,91]. The key effects of heat stress on selected crops are summarized in Table 3.

High temperatures affect photosynthesis and its components in rice [107], wheat [108], maize [109], beans [110], and the chickpea [111]. On the other hand, an increase in respiration, rather than photosynthesis, was conspicuous in bean genotypes in response to elevated temperatures [112]. Heat stress differentially affects the stability of various proteins, membranes, RNA species, and cytoskeleton structures, and it alters the efficiency of enzymatic reactions in the cell [58,113]. Every plant’s growth stage is susceptible to heat stress, but the reproductive stages are the most vulnerable. A slight increase in temperature during flowering may lead to a loss of grain yield. The failure of grain formation and development can be attributed to impaired pollen germination, pollen tube growth, and reduced ovule viability, as well as anomalies in stigmatic and style positions, a reduced number of pollen grains retained by the stigma, impaired fertilization processes, obstacles in the growth of the endosperm, and unfertilized embryos or embryo abortions as reported in rice [114], wheat [115], the chickpea [80] and other grain crops [59]. High temperatures can change nutrient uptake patterns as seen in pearl millet, which can accumulate more N, P, and K relative to unstressed plants, but the uptake of Ca^2+^, Mg^2+^, Na^+^, and S remains unaffected [116].

### 2.3. Salinity

Salinity is a significant abiotic stress that restricts crop growth and productivity and is characterized by an excessive concentration of soluble salts in the soil that suppresses plant growth in many irrigated, arid, and semi-arid regions of the world [117]. The extent of the salt injury depends on the crop species, cultivar, growth stage, any ecological factors, and the nature of the salts in the soil. The physiological and biochemical responses of plants under salinity stress are shown in Table 4.

An increase in the EC above 0.88 dS m^−1^ led to a decrease in rice grain yield [118]. The grain yield loss of wheat genotypes in response to salt stress was as high as 82% in controlled environmental studies [119]. Salinity can adversely affect seed germination in rice [120], wheat [121], maize [122], the faba beans [123], the chickpea [124], and the mung bean [125,126]. This is due to the high osmotic potential outside the seed, inhibiting the absorption of water, or due to the toxic effects of Na^+^ and Cl^−^. High Na^+^ concentrations prevent the absorption of K^+^ ions, which are highly essential for growth and development [127].

The interference of salts with the nutritional homeostasis of plants increases ionic ratios such as Na^+^/K^+^, Na^+^/Mg^2+^, Na^+^/Ca^2+^, Cl/H_2_PO_4_, and Cl^−^/NO^−3^, which adversely affects plant cellular processes [128,129]. Crop species and cultivars widely vary in their tolerance to salinity. For example, durum wheat is more sensitive to salt than bread wheat at critical growth stages such as at germination and early growth [130]. These differential responses of durum and bread wheat are due to differences in their ability to eliminate Na^+^ from the leaf and to discriminate between K^+^ and Na^+^. It is well-known that salinity affects plant growth through low soil solution osmotic abilities and nutritional imbalances [131]. In cereals, it alters plant growth through ionic imbalances, oxidative alterations, metabolic regulations, nutritional disorders, membrane disorganization, and low cell differentiation rates in crops like rice [132], wheat [133], maize [116], and the chickpea [134].

### 2.4. Waterlogging

Waterlogging and submergence causes substantial yield losses in food grain crops. Climate change scenarios predict increases in future incidences and intensities of floods, especially in the tropics and subtropics [144]. Most dryland cereals such as maize, wheat, and barley are sensitive to waterlogging, causing up to 20% yield losses in irrigated areas and even more significant losses in rainfed ecosystems exceeding 40% [145]. Damage estimated up to 100% may be caused by waterlogging stress, depending upon the crop, the length of the waterlogging, and the stage of plant growth. Based on the height of the water, the flood can be categorized as waterlogging when it is superficial and covers only the root, or it can be categorized as submergence when the water completely covers the aerial tissues of the plant [146].

Underwater plant cells that carry out photosynthesis do not readily exchange oxygen and CO_2_. Therefore, flood-damaged plants have a lower rate of aerobic cellular respiration than normal plants. Low CO_2_ concentrations in flooded leaves subsequently limit photosynthesis. Flooding leads to an energy crisis within the cells of plants [147]. Waterlogging leads to hypoxic or anoxic conditions in the soil, in which the soil becomes devoid of oxygen. The lack of oxygen for root respiration reduces the rate of root growth. Soil toxicity prevents root development and encourages root decay.

The waterlogging reduces photosynthesis due to stomatal closure, as well as abscisic acid (ABA), ethylene, and active oxygen species production. In addition, stomata closure often restricts CO_2_ in plant cells and induces the accumulation of oxygen free radicals. Plants under waterlogged conditions experience increased cellular damage from reactive oxygen species [148].

## 3. Combined/Multiple Stresses

Plants are constantly exposed to various environmental stresses such as salinity, drought, cold, and high temperatures. The impact of abiotic stresses on the grain yield of cereals and legumes is shown in Table 5. These multiple and combined stresses can vary in duration and intensity and can act simultaneously or sequentially. Earlier stress interactions have a significantly higher negative impact on crop productivity than each of the different stress components applied individually [149]. Drought and heat stress are excellent examples of two distinct abiotic stress conditions in the field simultaneously. This combination has a significantly greater detrimental effect on the growth and productivity in crop plants as compared to stresses applied individually [150]. Negative interactions have also been demonstrated in plants subjected to high intensity light and drought [151], high intensity light and cold stress [152], and drought and high temperatures [153,154].

In comparison, relative to each of the stresses applied separately, certain stress combinations may benefit plants. Examples include elevated CO_2_ levels, which are advantageous when combined with other stresses such as salt or high light [155]. Salinity in combination with heat stress in tomatoes enhances the protection against the damaging effects of salinity, suggesting that the accumulation of osmoprotectants such as glycine betaine and trehalose could play an important role in protecting plants against stress combinations. Combinations of drought and ozone could decrease the ozone intake in stomata by reducing stomatal conductance because of drought stress [156].

## 4. Mechanisms Associated with Stress Tolerance

Plants are constantly under pressure from environmental stresses, and they tolerate or resist stress by various adaption and acclimation mechanisms. Plants have evolved complex physio-biochemical and molecular strategies to neutralize the effects of abiotic stress [170,171]. The responses of plants to stress include changes in physiological processes such as photosynthesis, changes in ion levels, changes in membrane fluidity, the accumulation of osmolytes, the synthesis of secondary plant metabolites, phyto-chelation, the activation of ROS scavenging machines, and more. Broadly, there are two groups of stress-responsive genes which protect crop plants from abiotic stresses. One group includes regulatory genes, and the other group includes biosynthetic and structural genes.

### 4.1. Adaptations to Drought Stress

Plants adapt to droughts by adjusting their phenology, morphology, and physiology at the cellular and molecular levels. The drought-induced inhibition of growth as well as yield reductions can be attributed to adverse effects on plant functions and processes, particularly plant water uptake, water use efficiency, and the partitioning of biomass to grains.

#### 4.1.1. Escape Mechanisms

Plants, including cereals and legumes, tend to escape droughts by curtailing the crop growth duration or accelerating phenological phases, referred to as a flexibility in phenology [172]. This feature has been utilized to develop short-duration drought-tolerant crop cultivars (e.g., rice, wheat, sorghum, the pigeon pea, the peanut, the chickpea, and the lentil). However, severe and prolonged droughts can reduce the grain yields of these crops drastically. In plants that utilize escape mechanisms, seed or pollen germination usually occurs before acute water shortage. However, plants with growth plasticity seem to grow slower in the dry season with few flowers but have more fruits and seeds in the normal season.

#### 4.1.2. Dehydration Avoidance

The plant adjusts to droughts by lowering water loss and getting more water through root uptake. Adaptive traits are used to set the background of a “low transpiration rate in water-saving plants” and an “osmatic adjustment in water-spender plants” to prevent dehydration [173,174].

Plants mostly rely on leaf relative water content, osmotic adjustments, and root architecture to enhance the yield under drought stress [175]. Reducing the size of the leaves can be regarded as a mechanism to minimize water loss by transpiration. It has been documented that leaf shedding occurs from the oldest leaves to the youngest leaves during sequential water shortages, and drought-tolerant genotypes have higher leaf-shedding rates [176]. Closed stomata also suggest drought tolerance because in response to drought stress, stomata are closed, and transpiration is decreased [177]. Water loss is significantly influenced by the stomatal movement, stomatal density, and the resistance of plants to transpiration. Remarkable variations in the stomatal functions of various plants during droughts have been identified [178,179]. Adaptations to droughts can reduce the stomata size or the number of stomata. These unique anatomical features were created to protect the plant from harsh environmental conditions. Plants from arid and semi-arid habitats show sunken stomata, protected by resinous layers, waxes, and detritus on the laminae that sticks to stomata [180].

Drought-tolerant plants exhibit adaptive root properties, including long roots, high densities of roots, and intense rooting systems [177,181,182]. Plants selectively produce and extend their roots towards the wet part of the rhizosphere due to specific genes associated with this process [183,184]. Denser roots can absorb greater amounts of water because of the more extensive root system [185]. The gene responsible for root hair elongation is associated with drought tolerance in maize [186], rice [187], wheat [188], and grain legumes [5]. Drought tolerance is the plants’ ability to tolerate low tissue water content by adaptive traits, including preserving cell turgor by osmotic adjustment, preserving cell elasticity, and improving protoplasmic resistance. The antioxidative system that operates in response to abiotic stress can also contribute to dehydration tolerance.

#### 4.1.3. Osmoregulation

The cellular dehydration of tissues occurs when plants are exposed to extreme temperatures, droughts, and salinity. The plant cell produces osmolytes such as sugars, proteins, nucleic acids, and amino acids to protect from dehydration, as reported in wheat [189]. An osmotic adjustment is the process of solute accumulation mechanisms in plant cells when the water potential is limited, which helps maintain the turgor. The accumulation of osmotic substances is controlled by the intricate cellular processes involved in water flux and osmotic adjustment during abiotic stress conditions [190]. Sugars are critical biomolecules involved in various crucial physio-chemical mechanisms, from seed germination to senescence in cereals and grain legumes [191]. Sugars play diverse roles such as osmolyte biosynthesis, as well as maintaining membrane integrity, growth, and differentiation [192]. Proline is a compatible osmolyte that protects the cellular machinery from oxidative damage and maintains the homeostasis of photosynthesis [193]. Amino acid-derived compounds such as glycine betaine and polyamines also contribute to abiotic stress tolerance in various plants, including rice [194,195]. The accumulation of these osmolytes in the cytosol is an essential stress response to adjust the osmotic equilibrium in the plants under abiotic stress [194].

#### 4.1.4. Antioxidant System

Reactive oxygen species (ROS) such as singlet oxygen, hydrogen peroxide, superoxide, and hydroxyl radicals are involved in various cellular functions [196,197]. ROS, which exist at a low level under normal conditions, tend to increase when plants are exposed to stress. At high levels, ROS are toxic to cells, while the same molecule at low concentrations can function as a signal transducer that activates a local and systemic plant defense response against stress [198]. Chloroplasts, peroxisomes, endoplasmic reticulum (ER), mitochondria, and apoplasts after exposure to any stress may rapidly produce ROS, which are dangerous to the plant if not mitigated or scavenged. Plant peroxisomes are considered as a factory of ROS and a regulator of NO and H_2_O_2_ metabolism [199]. Rezayian et al. [200] reported that NO stimulates the antioxidant system and osmotic adjustment in the soybean under drought stress. Biswas [201] reported that ROS and reactive carbonyl species constituted a feed-forward loop in auxin signaling and play an important role in lateral root formation. Vanillic acid [202,203], selenium [102,204] and cerium [101] also play roles in antioxidative defense mechanisms in plants.

The plant possesses antioxidant machinery with the enzymatic and non-enzymatic components to mediate redox signaling and ROS homeostasis linked to acclimation responses to abiotic stressors. In response to stress, several antioxidative enzymes are produced by the plant. Superoxide dismutase (SOD), catalase (CAT), and peroxidases (POX) are among the enzymatic components of antioxidant systems that regulate the homeostasis of ROS within organisms, as reported in wheat [95,205], rice [206], sorghum [77,100,101], pearl millet [79] and the chickpea [6]. The non-enzymatic antioxidants include components such as ascorbic acids, α-tocopherol, flavonoid, glutathione, and carotenoids, which efficiently alleviate oxidative damage by reducing ROS activity or by working together with the enzymatic players to achieve efficient antioxidant activity via the utilization of H_2_O_2_ [207,208,209]. The ascorbate–glutathione pathway comprises of AsA, GSH, and four enzymes, viz. ascorbate peroxidase, monodehydroascorbate reductase, dehydroascorbate reductase, and glutathione reductase, which play vital roles in detoxifying ROS and ultimately mitigate oxidative damage in plants under abiotic stress [210].

At the cellular level, singlet oxygen, superoxide, hydroxyl ion, and hydrogen peroxide (H_2_O_2_) generation are typical heat stress incidents [211]. To defend against the damaging effects of the over-production of ROS under heat stress, plants have evolved complex antioxidant enzymes and non-enzymatic antioxidants, as reported in many crops such as wheat [61], the chickpea [212], and the pigeon pea [213]. The involvement of nitric oxide in ROS generation has been associated with abiotic stress tolerance [214].

#### 4.1.5. Phytohormones

Plant hormones play pivotal roles in controlling responses to several internal and external stimuli. Abscisic acid (ABA) is the key hormone considered to regulate the response of plants to abiotic stresses. Increased levels of endogenous ABA under drought stress conditions have been reported in many plant species, which include grain crops like sorghum [215], rice [216], barley [217], the soybean [218], and wheat [219]. The level of ABA is also influenced by cold stress in wheat [220], heat stress in wheat [115,221,222], and salt stress in maize [223]. ABA accumulates in stressed plants, interrupts their photosynthesis, and stimulates stomata closure to reduce water loss through transpiration. The roles of ABA in abiotic stress tolerance have been reported in a variety of plant species through its exogenous application either as foliar spray or as a seed primer in different crops, including cereals and legumes [224,225,226].

The exogenous application of ABA may also increase responses to droughts in wheat [227,228]. ABA is considered to have a beneficial impact on stress resistance after exogenous applications or by overexpressing genes due to its increased endogenous content in plants. ABA induces the expression of several genes whose products are essential for stress and tolerance reactions, such as osmo-protective synthesis enzymes [229]. Under drought conditions, ABA is synthesized in the roots and exported to shoots, as well as causing stomatal closure. The exogenous applications of auxin [230] and ethylene [231] are also effective in increasing abiotic stress tolerance; however, doses and stages are species-specific. Seed priming with auxin [232] and GA [233] was found to reduce the adverse effects of drought stress on yields, and was associated with improved physiological functions.

Cytokinin (CK) postpones the premature senescence of leaves and death during drought stress and promotes adaptive traits that help enhance grain yields. The increase in endogenous levels of CK through the expression of the CK biosynthesis gene isopentenyl transferase (*IPT*) delays cell senescence caused by droughts and improves crop yields [234]. In addition to controlling root growth and branching, CK inhibits the primary root growth and branching under drought stress [235]. Jasmonic acid also plays a vital role in abiotic stress tolerance, mainly in drought stress in plants [236].

### 4.2. Adaptations to High Temperature Stress

Plants adapt to high temperature stress through morphological and physiological adjustments. Mechanisms may vary across the crop growth stages. Critical growth stages such as anthesis and grain filling are highly sensitive to above optimum temperatures. Some adaptation mechanisms to cope with high temperature stress includes canopy cooling through transpiration, the involvement of heat shock proteins, various endogenous protectants, the antioxidant system, and the regulation of the biological clock, as reported recently.

#### 4.2.1. Transpirational Cooling

To cope with elevated ambient temperatures, plants transpire more water to maintain the requisite optimum and cooler canopies for physiological function. The mechanisms of transpirational cooling in response to high temperatures and its implications have been comprehensively illustrated [212,237,238,239,240,241,242,243,244,245,246]. This is an avoidance mechanism that allows the plant to function and maintain cooler canopies. However, this requires the availability or access to soil water resources and irrigation.

#### 4.2.2. Heat Shock Proteins

High temperature stress leads to the production of a group of proteins called heat shock proteins (HSPs), or stress-induced proteins. Plants under stress tend to produce less normal proteins and up-regulate genes associated with HSPs [206]. About 20 HSPs have been found in plants, and the diversification of these proteins reflects the adaptation or tolerance to heat stress. The general function of HSPs is to serve as molecular chaperones that control the folding and aggregation of proteins and the localization and degradation of all plants. As chaperones, these proteins avoid the irreversible aggregation of other proteins and engage in protein refolding under heat stress conditions [247]. The HSPs protect cells from damage and make them easier to recover after returning to normal growth conditions. Under high temperature stress, some high molecular weight HSPs, such as HSP101, were recognized as the important proteins for high temperature responses in crop plants like maize [248]. Low molecular weight HSPs, i.e., −18.1 and −17.9, were reported to accumulate in the pea while it was treated for four hours at 42 °C. The changing responses and expressions of the HSPs vary in different phases of development [206]. HSP90 also showed an increased expression under heat stress in rice and the soybean [249]. The involvement of HSPs have been reported in legume crops like the mung bean [250], common bean [251], and in the pigeon pea [252]. The crops where HSPs were involved in a high temperature response that was reported recently included rice [253], wheat [254], and the chickpea [255].

#### 4.2.3. The Role of Protectants

Exogenous applications of osmoprotectants, phytohormones, signalling molecules, and trace elements have shown positive impacts on plants grown under heat stress, as they have growth-promoting and antioxidant abilities. The heat tolerance in plants may be increased by the exogenous application of osmoprotectants [256]. Several different endogenous compounds have been found to be effective in moderating the intensity of heat stress in plants. Tocopherol, a key lipid-soluble redox buffer, acts as a scavenger of singlet oxygen species and other ROS and helps in mitigating heat stress in plants [257], as reported in rice seedlings [258] and wheat [259]. Ascorbate also has the potential to improve heat stress tolerance in maize [111,260]. The primary function of ascorbic acid is to prevent ROS activity and the photoinactivation of PSII and thus prevent the entire photosynthetic apparatus from damage [261]. Compounds like jasmonic acid can alleviate high temperature-induced spikelet-opening impairment during anthesis by enhancing antioxidant abilities and osmotic regulation as reported in wheat [262] and maize [263].

#### 4.2.4. The Role of ROS and Antioxidants

At the cellular level, singlet oxygen, superoxide, OH, and hydrogen peroxide (H_2_O_2_) generation and reactions are typical heat stress incidents [211]. The production of ROS damages the membranes of several organelles and structures of cells, making them dysfunctional. To defend against the damaging effects of the over-production of ROS under heat stress, plants have evolved complex antioxidant enzymes and non-enzymatic antioxidants as reported in wheat [61], sorghum [77,101,102], the soybean [85,105], the chickpea [212], the pigeon pea [213], and the moth bean [264]. The involvement of nitric oxide in ROS generation has been associated with abiotic stress tolerance [214]. Generally, there is a balance of ROS production and antioxidants under normal conditions. However, under stress conditions, the production of ROS is greater than the antioxidants, leading to the accumulation of ROS, which damages various membranes and limits the functionality of the cell.

#### 4.2.5. The Biological Clock

The temporal effects of thermal stress during the plant’s life cycle and the diurnal cycle are critical, and plants have mechanisms to sense peak periods of stress and exhibit appropriate adaptive strategies. Recently, it has been reported that high temperature stress can influence plants’ biological clocks, which affects genes associated with the plant’s time sensing mechanisms [265]. Wu et al. [266] reported the association of PePIF3a, a positive regulator in plants’ drought and salt stress responses, with circadian rhythms. The time of day of flowering or early morning flowering is a mechanism adopted by some crop species or genotypes to escape heat stress. These crops and genotypes flower early and complete the process of pollination and fertilization early in the morning or during the cooler times of the day before the daytime higher temperatures are reached [59,68,96,114].

### 4.3. Adaptations to Salinity Stress

Unlike high temperatures and droughts, stresses caused by salinity are not periodic. Plants must cope with the high level of salts in soil throughout their life cycle, though events of precipitation may change the adverse impact of salts depending on soil characteristics. Hence, plants have adaptive mechanisms for both osmotic stresses and the toxic effects of excess ions.

#### 4.3.1. Ion Transport and Homeostasis

Salinity contributes to two types of stress in plants. Osmotic stress occurs at the initial stage due to less water in the soil and the increased cytosolic Na+ and chloride in the matured leaves at later stages [267,268]. The maintenance of ion homeostasis by ion uptake and compartmentalization is essential for growth during salt stress, and ion transporters play a crucial role in this process in many crops [269], including rice [270], maize [271], wheat [272] and the chickpea [273]. Excess salt in the plant is partitioned into the cell vacuole or deposited in old tissues and is soon excreted from the plant to protect it from salt-related stress. The superiority of bread wheat over durum wheat has been attributed to the differential ability to sequester sodium in vacuoles in roots [274,275]. Under stressed conditions, the survivability of the plant depends upon the activity of V-ATPase [276], as shown in wheat [277] and barley [278].

The salt-sensitive species in crop plants, such as rice [279], cannot regulate Na^+^ transport at high salinity levels, where ionic effects dominate the osmotic effects. Plant cells need to maintain high K and low Na^+^ levels [280]. Thus, salinity stress tolerance requires maintaining osmotic homeostasis and ionic homeostasis. In general, to survive under high salinity conditions, the plant either adopts avoidance or tolerance mechanisms for osmotic homeostasis and ionic homeostasis. The main adaptive tolerance mechanisms for salinity stress involve successfully eliminating excess Na^+^ ions from the cytoplasm, and its accumulation within the vacuoles [131]. A central mechanism controlling the tolerance of plants to salt stress is the ion compartmentalization of different tissues and cells. Sequestering more Na^+^ in the root and flag leaf sheath in tolerant wheat genotypes can maintain lower Na^+^ concentrations with higher K^+^/Na^+^ ratios in photosynthetically active flag leaves [281]. Excess salt triggers the cytosolic Ca^2+^ concentration, which activates the Ca^2+^ binding proteins and upregulates the Na^+^/H^+^ antiporter to remove Na^+^ [282]. Intracellular Na^+^/H^+^ antiporters mediate the compartmentalisation of Na^+^ in cell vacuoles. The NHX is an antiporter that regulates the cell pH and preserves the homeostasis of Na^+^/K^+^ in plants. It also plays an important role in cell volume control which is needed for sequestering.

#### 4.3.2. Compatible Solutes

Under salinity stress, plants can synthesize compatible solutes to ensure their survival. These compounds include glycine betaines, amino acids, polyols, non-reducing sugars, and polyamines [283]. Some amino acids such as cysteine, arginine, and methionine decrease in a salty environment while proline levels increase. The accumulation of proline is a well-known process to relieve salinity stress. Intracellular proline not only provides resilience to stress but also plays a crucial role in stress recovery. The modulation of the proline metabolism for tolerance to salt and droughts has been reported [284]. It has been documented that reduced forms of sugar, such as glucose and fructose, serve as osmoprotectants under salinity stress. Glycine betaine allows for variations in promoting the alteration of osmoticum by discriminating against Na^+^/K^+^, thereby preserving induction and retaining membrane stability, which significantly reduces sensitivity to salinity [285]. Munns et al. [286] have comprehensively reviewed osmotic adjustment mechanisms and energy requirements for driving this process. Small molecules such as melatonin have been reported to play a critical role in salt stress tolerance in plants [287,288].

### 4.4. Adaptations to Excess Submergence and Waterlogging

Plant partial/complete submergence and waterlogging restricts oxygen diffusion to submerged tissues and inhibits aerobic respiration [289]. The decreased oxygen triggers the cessation of the tricarboxylic acid cycle and oxidative phosphorylation. Consequently, the primary source of ATP production shifts from the mitochondrial electron transport chain (ETC) to ethanol fermentation. However, the efficiency of ATP production from ethanol fermentation is lower than that of the ETC. Upon reaeration after a period of oxygen deprivation, ethanol trapped in tissues will be converted to acetaldehyde, causing post-anoxic cell injuries. Furthermore, the concentrations of potentially toxic compounds increase in anoxic soils, and these can enter through roots, damaging both root and shoot tissues. The ROS also accumulate excessively upon oxygen deprivation or re-oxygenation under light conditions [290,291]. Antioxidant enzymes, including superoxide dismutase, catalase, and various peroxidases can effectively reduce ROS activity. Other non-enzymatic components of antioxidants such as ascorbate, glutathione, and β-carotene also play an important role in removing toxic oxygen compounds [290]. Despite these complex sets of detrimental effects posing challenges to the plant in flooded soils, some progress is being made in developing flood-tolerant varieties of cereals, particularly in rice.

Plants have many defensive mechanisms to defend themselves against waterlogging stress, such as forming airspaces (aerenchyma) in the root cortex, expanding the stem (hypertrophy), forming adventitious roots near the soil surface, and root tip death [292]. Rice has developed specialized anatomical and morphological traits such as aerenchyma, radial oxygen loss barriers, adventitious roots, and the ability to form a leaf gas film to adapt to excess water conditions. However, these strategies are insufficient for survival under continuous and complete submergence, which leads to death due to oxygen starvation. Some Asian rice varieties have further developed additional traits such as aerobic germination, the quiescence of leaf elongation in response to flash floods, and internode elongation under periodic flooding to overcome prolonged submergence [293]. Plants can get their leaves out of the water by growing the shoot above water. This ‘escape technique’ can be accomplished by high growth in stems, as observed in rice [294]. The possibilities of the modulation of the fermentative and sucrose metabolizing pathways under waterlogging conditions and the genetic variations in these mechanisms have been reported [295].

## 5. Explored Mechanisms of Abiotic Stress Tolerance for Crop Improvement

In plants, abiotic stress tolerance is a complex trait involving many different metabolic pathways and cellular and molecular components. Abiotic stresses commonly induce various responses at the morphological, physiological, biochemical, and genomic levels. For several decades, the plant research community has amassed a highly comprehensive understanding of the physiological and biochemical mechanisms that facilitate productivity maintenance in response to several abiotic stresses like droughts, flooding, heat stress, cold, salinity, and heavy metals. Understanding the abiotic stress tolerance mechanisms laid the foundation for the development of climate-resilient crop varieties [296]. The conventional breeding approaches have randomly exploited these plant tolerance mechanisms with limited success. Conventional breeding approaches are limited by the complexity of stress tolerance traits and the lowered genetic variation exhibited by most crops due to domestication bottlenecks. Furthermore, abiotic tolerance mechanisms in crop plants are limited and have largely failed to bridge the gap between theoretical research and crop breeding. Therefore, unraveling the genetic, epigenetic, transcriptomic, and metabolomic bases of stress tolerance mechanisms/traits is crucial for breeding climate-resilient or abiotic stress-tolerant crop varieties [297]. However, some success has been achieved in understanding the crop tolerance mechanisms to abiotic stresses, and a few of them have been explored for crop improvement. Some of the explored mechanisms involved in different methods of abiotic stress tolerance were compiled and presented in Table 6.

### 5.1. Early Flowering

Early flowering or maturity (EF/EM) is the most critical phenological trait/mechanism exploited by breeders for the development of short-duration varieties which can escape abiotic stresses, particularly droughts and heat stress. Early flowering and seed set before an upcoming drought event are important in legumes [330] and cereals [298]. This trait is controlled by three groups of genes, vernalization (*Vrn*), photoperiod (*Ppd*), and earliness per se (*Eps*), and the genetics of these traits have been studied extensively, particularly in cereals [331,332,333,334]. Shavrukov et al. [298] gave an insight into the early flowering mechanisms and discussed drought escape, with wheat as a target crop. Several studies reported that the crops with EF/EM traits could produce higher and more stable yields under drought conditions [331,335,336]. Furthermore, this is also supported by reports of more seeds under water limitations of EF/EM in pearl millet and sorghum [336,337]. Four cultivars of the chickpea and seven mutant mung bean lines flowered 2–4 weeks earlier than traditional cultivars and parental forms, respectively, displaying an enhanced seed yield [336].

Matyszczak et al. [299] identified two near-isogenic lines, *BW507* and *BW508*, in barley which were reported to carry two independent early-flowering mutant loci, mat-b.7, and mat-c.19, respectively. They mapped the mutation in *BW507* to a 31 Mbp interval on chromosome 2HL and concluded that *BW507* has a deletion of Mat-c, which is an ortholog of *Antirrhinum majus* CENTRORADIALIS (*AmCEN*) and *Arabidopsis thaliana* TERMINAL FLOWER1 (*AtTFL1*) and is a key gene in regulating early flowering. There is evidence that evolution can favor EF/EM traits in native populations of plants even without the pressure of oncoming drought stress [298]. Zonneveld et al. [338] concluded that during evolution, *Vigna taxa* more easily acquired phenological traits for a short life cycle to escape droughts and heat stresses compared with acquiring physiological traits.

### 5.2. Root System Architecture

Variations in root system architecture can be explored for the development of climate-resilient and abiotic stress-tolerant crops by improving water and nutrient use efficiency [339]. The primary root elongation rate and ABA accumulation under different water conditions have been studied in 12 maize inbred lines to assess the relationship between root growth and hormonal conditions [340]. Histograms of primary root elongation responses to the varying water deficits suggest multiple mechanisms may be responsible for the response to water stress observed in different maize lines [340]. A recent study on wheat revealed wide variability in root system architecture and shoot traits at the seedling stage in an association panel [341]. The root diameter and the distribution of the metaxylem vessels contribute to drought tolerance in legumes [342].

Phule et al. [343] observed that the formation of fewer aerenchyma, thickened roots, and larger xylem areas were critical anatomical traits associated with aerobic adaptation compared to anaerobic conditions. The photosynthetic rate was significantly higher in the rice cultivar CR Dhan 202 than BPT 5204 under aerobic conditions. The morpho-physiological results showed that the root length, total dry weight, and the photosynthetic rate are the key parameters for aerobic adaptation. These root anatomical and morpho-physiological traits associated with the adaptation can be used as screening criteria for the phenotyping and selection of genotypes suitable for the aerobic cultivation system. This information on morpho-physiological traits is expected to expedite the development of aerobic rice varieties in aerobic breeding programs.

Polania et al. [344] identified seven standard bean lines (SEA 15, NCB 280, SCR 16, SMC 141, BFS 29, BFS 67, and SER 119) that showed greater root vigor under drought stress in the greenhouse, and higher values of grain yields during drought stress under field conditions. Such water use-efficient plant ideotypes (water-spender ideotypes represented deeper root systems, while the-water saver ideotypes showed a relatively shallower root system) could serve as parents for improving drought tolerance in the common bean.

### 5.3. The Role of Chloroplasts in Plant Abiotic Stress Responses

Photosynthesis in higher plants is sensitive to various abiotic stresses. The photosystem II (PSII) is the most sensitive to desiccation. Its sensitivity could be strongly associated with the desiccation severity [345]. Chloroplasts are semiautonomous intracellular organelles central to photosynthesis and are essential for plant growth and yields. The significance of the function of chloroplast-related genes in response to climate change has not been well studied in crops. The metabolites synthesized in chloroplasts protect plants from abiotic and biotic stresses, including heat, cold, drought, salt, light, and pathogens [346,347]. Through meta-expression analyses under abiotic stress conditions, Yoo et al. [348] identified 264 cold or heat stress-responsive plastid-related genes in rice. Furthermore, the functional characterization of plastid-related genes emphasized the significance of genes for crosstalk between chloroplast development and heat stress. They concluded that chloroplast-related genes affected the abiotic stress response mainly through the high temperature response, with little effect on responses to droughts and salinity stress. Furthermore, they predicted a protein to protein interaction network analysis associated with high temperature stress which is expected to provide the basis for studying molecular mechanisms by which chloroplasts will respond to different abiotic stresses under changing climatic scenarios. Khurana et al. [349] characterized the chloroplast localized wheat membrane protein (TaRCI) and its role in heat, drought, and salinity stress tolerance. This membrane protein (TaRCI) could be a potential candidate for gene manipulation for improving stress tolerance in crop plants in general, and wheat crops in particular.

### 5.4. Deficit and Excess Water Stress Tolerance

In recent years, the use of root system architecture for the improvement of stress tolerance in crop cultivars has gained attention. The discovery of the deep root 1 (*Dro1*) gene [187] has been utilized for improving drought tolerance [303]. Similar strategies with homologs of deep root genes are being explored for other cereals such as wheat [350]. The utility of *Dro1* homologs in the improvement of salt tolerance in rice has been demonstrated [351].

The physiological and molecular responses of rice to flooding have been extensively studied [315], providing evidence for several traits associated with submergence tolerance. The most progress was the discovery and deployment of the SUBMERGENCE 1 (*SUB1*) locus in rice, conferring tolerance to complete inundation (submergence) [308,352]. *SUB1A* was identified and cloned from a submergence-tolerant cultivar, i.e., FR13A, and it had been developed through selection from farmers’ variety “Dhallaputia” grown in Odisha (India). *SUB1A* is the dominant gene(s), and fine mapping of this gene on chromosome 9 has been completed. A marker-assisted backcross breeding approach is now being successfully exploited for the development of high-yielding submergence-tolerant rice cultivars. Under mild stress (5–6 days submergence), plant mortality in rice is generally very low, yet extensive leaf damage occurs. However, the damage is almost nil in cultivars with *SUB1 QTL* genes due to the maintenance of higher activities of antioxidant enzymes. Prolonged water stagnation decreases the grain yield in cultivars with *SUB1* (Swarna-*Sub1*) compared to those without *SUB1* (e.g., Swarna). Sarkar et al. [353] suggested that Swarna-*Sub1* is suitable for flash flood conditions. They also concluded that the maintenance of chloroplast integrity could be a better option for predicting the plant survival under submergence. Some researchers believe that the ideal combination for an adaptation to complete flooding is submergence tolerance (survival underwater) together with some elongating abilities [353].

The geographical distribution of these accessions harboring *SUB1A-1* suggests that *SUB1A-1* from wild species might have introgressed around the Ganges Basin and subsequently spread to other areas of South Asia [354]. By contrast, *SUB1A-1* is absent in submergence-tolerant accessions of wild rice with the CC genome (*O. rhizomatic* and *O. eichingeri*) and the CCDD genome (*O. grandiglumis*) [355,356], suggesting the presence of a *SUB1A*-independent mechanism in these rice species. Future elucidation of the *SUB1A*-independent mechanism may contribute to the future breeding of cultivated rice with strong flash flood tolerance.

A recent significant discovery of the cloning of the Leaf Gas Film 1 (*LGF1*), a wax synthesis gene involved in the leaf hydrophobicity and formation of the gas films necessary for gas exchange and underwater photosynthesis, is another important step towards developing flood-tolerant varieties [311]. There is evidence that cuticular wax accumulation is associated with drought tolerance in wheat [232], maize [357], and beans [358]. The dynamics of wax accumulation in leaves of wheat have been recently elucidated [359]. The discovery of *LGF1* briefly gave insights into the potential shoot and root traits that can improve submergence tolerance in rice. Kuroha and Ashikari [293] reviewed and discussed the recent progress in understanding the various molecular mechanisms and genetic factors regulating flooding tolerance in rice.

## 6. Unexplored Mechanisms and Genes Modulating Abiotic Stress Tolerance

An in-depth understanding of stress sensing, signal transduction, and the generation of the stress response are required to develop resilience to multiple abiotic stresses in grain crops, including major cereals and legumes. Uncoupling the molecular processes associated with stress tolerance is crucial for generating climate-resilient crops. It is crucial to differentiate the plant responses observed under a particular environmental stress factor and responses observed under a combination of stresses for developing climate-resilient crops [360]. Hence, there is an urgent need to include stress combinations while studying the plant responses to abiotic stress, employing molecular genetics, molecular breeding, or molecular physiology. Another challenging task is to link the biological processes at different scales [360]. The crops in silicon initiatives, wherein multi-scale models try to generate new concepts, prioritize bioengineering efforts in plant research [361]. The challenge is to build developmental models that fit well with the biological traits of the crop species and explain the plant responses to changes in the environment [360]. Multi-level models incorporating data from genetic, epigenetic, and transcriptional studies, along with data regarding splicing and post-transcriptional regulation, are likely to provide new insights into plant molecular responses under abiotic stresses [362]. The integration of machine learning algorithms with transcriptomic data and high-throughput phenotypic data is now essential to accelerate gene discovery processes, including genome annotation and gene regulatory network predictions [363].

Several traits associated with multiple abiotic stress tolerances are encoded by genes that were lost during cultivation. Large numbers of genetic resources of crops with diverse genetic variations are available; hence, there is ample scope to recover traits associated with resilience to abiotic stresses. Advances in genomics, molecular genetics, and phenomics, coupled with new methods for capturing genomic regions associated with abiotic stress tolerance, could be promising for attaining climate resilience in cereals and legume crops. The pyramiding of genes into a single background is now a viable strategy. Assembling appropriate gene combinations in elite varieties is a challenging task. Addressing the yield loss due to abiotic stress requires innovative technologies like genome editing and epigenetic modification. Combining genetic resources and transformative technologies from genome editing to synthetic biology could be helpful strategies to identify traits associated with tolerance to a multitude of abiotic stresses. Some novel opportunities are discussed in this section.

### 6.1. ABA Receptors

ABA is a master controller of transpiration and regulates ion channels and the expression of genes associated with abiotic stress tolerance in plants. ABA interacts synergistically or antagonistically with salicylic acid, jasmonic acid, and ethylene to regulate responses. The plasma membrane-localized G protein-coupled receptor (GPCR) and type G proteins (GTGs) [364] were considered ABA receptors. However, their targeted roles in signaling pathways and conservation in grain crop species remain characterized. The genetic manipulation of ABA signaling can be useful. The ABA signaling components, comprising of the ABAR–ABA–PP2C complex and SnRK2s, control ABA-mediated stomatal closure via ion transportation in guard cells, and there are transcriptional regulations required to develop stress responses. Further studies need to be carried out to illustrate the ABA signaling pathway components and effector genes. The PYL-ABAR gene family members differ in their tissue-specific responses, stress-responsive expressions, dimerization, and binding capacities to ABA. Hence, the physiological relevance of different combinations of ABAR–ABA–PP2C needs to be elucidated.

### 6.2. Engineering Orthogonal Receptors

An orthogonal receptor is an engineered receptor that can bind specifically to a synthetic ligand, which cannot interact with the natural receptor. The orthogonal receptor is not activated by the basal level of the endogenous ligand. The orthogonal receptor is helpful since they get activated by synthetic ligands at low concentrations, while the natural receptors require relatively higher doses of synthetic ligands for activation. The orthogonal receptor can be used to design crops for resilience to multiple abiotic stresses, where synthetic agonists or antagonists can be used to induce a specific physiological process such as metabolite production and tolerance to abiotic stress [365]. These studies may be crucial to engineer and use orthogonal receptors for enhancing stress tolerance.

### 6.3. Novel Transcription Factors

Plants have evolved sophisticated stress response strategies and harbor genes that encode transcription factors (TFs) to regulate the expression of stress-responsive genes. TFs could be candidates for enhancing resilience to multiple abiotic stresses. Recently, the TF modulation and overexpression approaches have been employed in crop plants; however, the diversity of TFs largely remains unexplored. TF families such as NAC, MYB, WRKY, bZIP, and ERF/DREB have been successfully characterized for their roles in eliciting abiotic stress responses. About 10% of genes encode TFs, which play an important role during different stages of the plant life cycle for a specific function [366]. Therefore, elucidating the mechanisms of the actions of TFs is crucial to explore the mechanism associated with plants’ responses to various abiotic stresses. There is the potential to engineer crops with TFs to enhance their tolerance to many stresses and characterize TFs for their role in stress tolerance in plants [367].

Advanced technologies like chromatin immunoprecipitation with massively parallel sequencing (CHIP-Seq) and next-generation sequencing (NGS) could be beneficial for genomic region identification for deciphering the role of TFs. The CRISPR/Cas (Clustered Regularly Interspaced Short Palindromic Repeats/CRISPR-associated protein) gene-editing tool can be used to modulate TFs. In addition, the functional redundancy of TFs needs to be addressed. Although previous analyses of TF overexpression in response to a specific stress have been very informative, studies are required to investigate whether the overexpression of stress-related TFs in transgenic plants enhances stress tolerance and growth without any yield penalty. Further studies are required to understand the mechanisms of the action of several TFs and their role in enhancing stress tolerance in food grain crops.

## 7. The Metabolic Control of Resilience to Abiotic Stress

The molecular breeding and molecular genetics for the genetic enhancement of plants for yield stability under adverse environmental conditions could potentially capture the effective resilience to abiotic stress. Plants generally reduce vegetative growth and accelerate reproductive development under stress. The genetic variations and underlying mechanisms that enable drought-resilient plants to conserve soil moisture and delay the accumulation of biomass until grain filling largely remains unclear. Higher yields under well-watered conditions and under a moderate drought at the time of flowering were achieved in corn that expresses a metabolic enzyme that converts trehalose-6-phosphate (T6P) to trehalose in the phloem companion cells at the base of the ear and in developing florets [368]. The modulation of T6P facilitates the photosynthate’s mobilization to the unfertilized floret and prolongs the photosynthetic activity of leaves during grain filling. Thus, novel genetic variations and genetic enhancements can achieve the integration of the metabolism and stress resilience to improve crops for yield stability under adverse environmental conditions.

## 8. Engineering Plants for Biomass Production under Abiotic Stress Conditions

Recent advances in molecular genetics and genomics have enabled the identification of a complex signaling network associated with plant growth and development. Many genes have been identified and characterized for their role in abiotic stress tolerance, employing genomics and molecular genetics. However, efforts should be made to unravel the crosstalk between the transcriptional circuitries for biomass production and abiotic stress responses. This knowledge could serve as a valuable resource to eventually custom design the crop plants for higher biomass production with less water use in a more sustainable manner under adverse environmental conditions.

The adaptation of plants to various abiotic stresses is a coordinated response involving many genes and their interactions with various environmental factors during the entire life span of crop plants [369,370]. Accordingly, a thorough understanding of the molecular responses in plants is crucial for improvements in plant biomass or yields. Advances in molecular genetics, genomics, tissue-specific or developmental stage-specific gene expression, and gene pyramiding can be promising in enhancing the photosynthetic efficiency in plants, contributing to a higher biomass.

Plant cell wall polymers form a significant component of plant biomass. The composition and amount of these polymers in the cell wall change with the developmental stage of plants and in response to stress conditions [371]. There is a need to delineate the genes associated with the biosynthesis of different cell wall components. In addition, manipulating endogenous plant hormones will further widen the scope of improving stress tolerance and biomass production. Integrating the key regulatory genes and transcriptional regulations of secondary cell wall biosynthesis through the cascade of activators and repressors could be crucial for designing crop plants with enhanced an biomass under stress conditions.

## 9. Future Perspectives and Conclusions

The challenge of improving abiotic stress tolerance in crops must be addressed with an understanding of the underlying complexities and with care taken to avoid grain yield penalties resulting from the introgression of relevant traits. A feasible approach to addressing this challenge should include the grain yield and the traits specific to targeted agro-ecologies. Although the possibility of improving stress tolerance has been successfully demonstrated through transgenic approaches, breeding varieties tolerant to abiotic stress is difficult. This has been attributed to the underlying complex mechanisms that influence the relevant traits and associated genes, which often act in coordination. There is ample scope for integrating different omics strategies to increase plant stress tolerance substantially, as marker-assisted selections by employing stress-related genes and QTLs are becoming more routine activities in the crop breeding program. Advanced tools such as CRISPR/Cas techniques for the modification of genes are becoming more relevant for the genetic improvement of abiotic stress tolerance in plants.

Recent advances in omics approaches, including genomics, proteomics, and phenomics have provided new opportunities for understanding abiotic stress responses in plants at a different scale, from the cell level to the whole plant level. Designs for stress-tolerant crops can be substantially improved with the additional insights into the mechanisms of stress tolerance in plants obtained through deep sequencing technologies and other omics approaches, such as metabolomics (Figure S2). Information emerging from epigenetics can be useful in understanding the mechanisms of the stress-memory of plants. Advances in molecular breeding methods can accelerate the use of functional molecular markers in marker-assisted selection. In addition, genomic tools can assist in understanding the gene networks associated with plant stress.

The existing knowledge gaps that are hindering the application of omics for abiotic stress tolerance in crop plants must be bridged through a system biology approach. Tools and methods for high throughput phenotyping must be optimized for the identification of relevant genes and their utilization in breeding different crops. This is likely to be aided by the emerging tools for extensive data analyses, which employs machine learning algorithms. Conventional approaches to crop production are now being complemented by remote sensing tools that are integrated for crop stress monitoring. Advances in imaging and sensor technologies, high throughput phenotyping, and remote sensing tools can enable rapid field-scale estimations of plant health and plant stress susceptibility or tolerance, guiding crop management decisions under an abiotic stress environment. The systematic evaluation of extensive germplasm collections to identify tolerant genes and genotypes, and the development of targeted breeding programs to enhance abiotic stress tolerance using traditional and novel methods, are both essential for increasing the yield and stress tolerance of food grain crops.

## Figures and Tables

**Table 1:** Effects of drought stress on yield of different cereal and legume crops.

Crop	Stress Description	Yield Losses (%)	Reference
Wheat	~40% water deficit	20–25	[28,29]
	No irrigation at reproductive and grain filling stages	30-32	[30]
	The different deficit moisture level	25	[31]
Rice	Soils dried beyond −20 kPa	23	[32]
	Withholding water at flowering (−30 ± 5 kPa)	23–24	[33]
	Moderate to severe stress at flowering	51–60	[34]
	Drought, water stress (~40% water deficit)	>50	[29]
	The different deficit moisture level	25	[31]
Maize	-40 and -80 kPa during flowering and grain filling, respectively	34–66	[35]
	50% FC at tasselling stage	20	[36]
	Progressive drought at vegetative stages	19–26	[37]
	Progressive drought at reproductive stages	42–47	
	Different irrigation regimes	34–66	[35]
	Drought with approximately 40% water reduction	39.3	[28]
Barley	Water stress (20% and 60% FC) during grain filling	50–60	[38]
	Drought stress at the start of anthesis (Field capacities 30%)	42	[39]
Pearl millet	Early drought stress from 3 weeks after germination for four weeks	>50	[40]
Millets	Rainfed conditions associated with terminal drought	53	[41]
Chickpea	Withholding water at reproductive stage	30–40	[42]
	Withholding water at early podding	80–90	[43]
	Under rainfed conditions with lifesaving irrigation	27	[44]
Beans	Withholding water after 25 days	80	[45]
Pigeon pea	Drought at flower initiation, soil moisture reduced from field capacity of 16% to 5.6%	11–40	[46]
Soybean	Rainfed in comparison to fully irrigated	33	[47]
	4 days of moisture stress during seed filling stage (R4–R6)	39–45	[48]
Black gram	Irrigated to FC when the weight of each pot reached 50% of FC	23	[49]
Mung bean	Withholding the irrigation at blooming stage to maturity stage and seed filling stage	51–85	[49]

**Table 2:** Physiological and biochemical responses of plants under drought stress.

Crop	Stress Description	Trait/Organ Affected/Impact	Reference
Rice	Soils dried beyond–20 kPa	Yield loss~22.6	[32]
Wheat	Drought, water stress (~40% water deficit)	Yield loss~25	[29]
Maize	5 days of drought stress at the V9 stage and 5 days after pollination stage by maintaining 14.0–15.0% SWC	Reduced kernel size, reduced expression of photosynthesis genes, and reduced yield	[50]
	Short-duration water deficits during the rapid vegetative growth period	28–32% loss of final dry matter weight	[51]
Sorghum	Season-long drought stress	Decreased harvest index, seed numbers, and seed size	[52]
Millets	Irrigation with mannitol (200, 400, and 600 mM) for 21 days at an interval of three days	Decreased germination, RWC; chlorophyll content increased root growth, proline, and MDA content	[53]
Chickpea	Drought, water stress for 3 weeks (40% of FC) at vegetative and flowering	Decrease in relative chlorophyll content, RWC; accumulation of H_2_O_2_	[54]
Pigeon pea	20 days at flowering and pod setting	Flower drop and decreased flower to pod conversion	[46]
Black gram	40% of field capacity	Reduced plant growth, branches, pod numbers, shoot and root dry weight, rate of photosynthesis and transpiration, stomatal conductance	[55]
Soybean	Withholding irrigation at critical stages	Reduced shoot biomass and seed yield, fewer seed pods, and seeds	[56]
Bean	Withholding irrigation after 25 days in field conditions	Reduced leaf area index, harvest index, pod partitioning index	[45]

**Table 3:** Effects of high temperature stress on different crop species.

Crop	Temperature	Growth Stage	Effect	Reference
Rice	40 °C	Emergence	Delay and decrease in the emergence	[92]
Wheat	45 °C		Reduced chlorophyll, photosynthesis, protein synthesis	[71,93,94,95,96]
	30/25 °C, day/night >32/22 °C, day/night		Green leaf area and productive tillers/plant reduced Decreased photosynthesis, membrane damage, floret fertility, seed numbers, seed size	
Maize	35/27 °C in day/night 14 days before reproductive to silking stage		Decreased cob weight, low sugar content	[97]
Sorghum	40/30 °C, day/night; 38/28 °C		Lipid peroxidation of chloroplast and thylakoid membranes; decreased floret fertility, grain weight	[23,77,78,98,99,100,101,102]
Pearl millet	>36/22 °C day/night	Emergence to maturity Booting to maturity	Decreased days to flowering, seed yield, and seed size; decreased pollen germination, numbers of seeds per panicle, and seed yield per panicle	[79]
Finger millet	>36/22 °C day/night	10 d after emergence through maturity	Decreased plant height, tillers, seeds per fingers, and grain yield	[73]
Chickpea	Gradual 29/16 C to 40/25 °C	Flowering	Lower pollen production, % pollen germination, pod set, and seed numbers	[103]
Black gram	40 °C	Flowering and pod setting	Reduced yield	[82]
Green gram	40 °C 60 days	Reproductive	Reduced yield	[83]
Common bean	32/25 °C	V4 until physiological maturity	Increased photosynthesis, conductance, and leaf area	[104]
	>28/18 °C	Emergence to maturity	Decreased seed-set, seed number per plant, seed number per pod, seed yield, and total dry weight per plant	[84]
Soybean	38/28°C (day/night), 14 days		Lower photosynthesis, stomatal conductance (gs), damaged membranes (chloroplast, thylakoids, mitochondria), and increased leaf senescence	[85,105,106]
Peanut	>32/22 °C	Flowering	Decreased fruit-set, pollen production, pollen viability, and pod numbers per plant	[86,87,88]
	>32/22 °C	Emergence through maturity	Decreased pollen viability, seed-set, seed number pod, seed size, and harvest index	[89]

**Table 4:** Physiological and biochemical responses of plants under salinity stress.

Crop	Salinity Level	Effect	Reference
**Rice**	EC 10 dS/m	Decreased root and shoot length	[135]
Wheat	100 to 175 mM NaCl	Reduction in spikelets per spike, delayed spike emergence and reduced fertility	[136]
Maize	1, 50, 100 mM NaCl	Stunted growth, reduced chlorophyll fluorescence, and enhanced levels of reactive oxygen species and 1,4-benzoxazin-3-one aglycones (aBX)	[137]
Millet	100, 200, and 300 mM NaCl	Depression in germination percentage, shoot and root growth rate, leaf relative water content, chlorophyll content, leaf K+ concentration	[138]
Chickpea	0, 4, 6, and 8 dS m^−1^	Reduced dry matter accumulation in root and shoot	[139]
Pigeon pea	0.5 to 4.3 dS m^−1^	Height, biomass, SSL, and RGR linearly decreased	[140]
Black gram	150 and 225 mM of NaCl	Reduction of leaf, shoot, and root biomass	[141]
Green gram	50 mM and 75 mM NaCl	Reduction in plant height, total chlorophyll, carotenoid contents, plant length, leaf area, rate of photosynthesis, yield characteristics	[142]
Common bean	100 mM NaCl	More lipid peroxidation, electrolyte leakage, abscisic acid (ABA); lower seed germination percentage, seedling growth, cell membrane stability index, and relative water content	[143]

**Table 5:** Impact of abiotic stresses on grain yield of cereals and legumes.

Stress	Growth Stage	Crop	Details of Abiotic Stress	Decrease in Yield (%)	Reference
High temperature	After heading	Wheat	>31 °C	16–25	[157]
			Delayed sowing in the field Minimum temp. 15–21 °C Maximum temp.−31–36 °C	22	[158]
	Heading	Rice	Diurnal temp 24–32 °C (control) 26–39 °C (high temp.)	21–55	[159]
	Tasseling stage	Maize	28/20 °C (control) 38/30 °C (high temp.) for 15 days	7–17	[36]
	Emergence to maturity	Sorghum	32/22 °C to 36/26 °C 32/22 °C to 40/30 °C	10 99	[160]
	Booting to start of seed filling	Pearl millet	28/18 °C to 36/26 °C 28/18 °C to 40/30 °C	50 98	[79]
	Emergence to Maturity	Finger Millet	32/22 °C to 36/22 °C 32/22 °C to 38/28 °C	75 84	[73]
	Emergency to Maturity	Chickpea	<32 °C/20 °C (control, normal sown) >32 °C/20 °C (high temp, late sown)	19−56	[81]
	Reproductive stage	Lentil	38/23 °C	85–88	[161]
	Reproductive stage	Mung bean	>40/25 °C	35–40	[83]
	Emergence to Maturity	Kidney Bean	>28/18 °C to 40/30 °C	6.5% per 1 °C	[84]
	Flowering	Peanut	36−44/26−34 °C	14−90	[89]
Salinity	Vegetative	Mung bean	50 mM and 75 mM NaCl	41−75	[142]
	Throughout crop duration	Wheat	0−200 mM NaCl	25−70	[162]
	Throughout crop duration	Faba bean	0.7, 3.0, and 5.0 dS m^−1^	27−47	[163]
	Throughout crop duration	Chickpea	0.7, 3.0, and 5.0 dS m	40−56	[163]
		Rice	3.8 to 6.4 dS m^−1^	~50	[164]
	Seedling and reproductive	Rice	4 dS/m^2^	28.8	[165]
Waterlogging	Vegetative or reproductive	Wheat	Early or late waterlogging for 14 days	14−29	[166]
	Vegetative or reproductive	Barley	Early or late waterlogging for 14 days	15−21	[166]
	Vegetative	Oats	0−35 days	79−83	[167]
	Vegetative/heading	Wheat	Flooding	30.4−39.4	[168]
	Vegetative or reproductive	Field pea	Early or late waterlogging for 14 days	94	[166]
	Seedling (V3), jointing (V6), and tasseling (VT) stages	Maize	Waterlogging (3, 6, and 9 days) and subsurface waterlogging (5, 10, and 15 days)	61.5−80.5	[169]

**Table 6:** Explored mechanisms involved in tolerance to different abiotic stresses.

Mechanism/Traits	Genes/Proteins/Enzymes and Other Molecules Involved	Target Crop	Abiotic Stress	Reference
Early flowering	Vernalization (Vrn), photoperiod (Ppd), and earliness per se (*Eps*) genes; *VRN1* and *Ppd-D1*	Wheat	Drought	[298]
	Mutant BW507 line (mutant allele Mat-c)	Barley	Drought	[299]
Osmoprotection and osmotic adjustment	Sugars (glucose, fructose, fructans, and trehalose)	Rice	Salinity	[300]
	Raffinose family oligosaccharides (RFO)	Rice	Cold and Drought	[301]
	γ-aminobutyric acid (GABA)	Wheat	Salinity	[302]
Lignin production (cell wall integrity)	*GmRD22* (regulates cell wall peroxidases and hence strengthens cell wall integrity under stress conditions)	Soybean and rice	Salt and osmotic stresses	[303]
Scavenging of ROS: Antioxidant Regulation				
	Enzymatic antioxidants: Catalase and pyrroline-carboxylate synthetase (*P5CS*), and sustained activities of superoxide dismutase (*SOD*) and ascorbate peroxidase (*APX*)	Chickpea	Salinity	[292]
	Non-enzymatic antioxidant compounds: Helicase proteins (e.g., DESD-box helicase and OsSUV3 dual helicase), Ascorbate, Glutathione	Rice Pea	Salinity	[304,305,306]
Flash flood tolerance	*SUB1A-1* encoding *AP2/ERF* (ethylene response factors), family transcription factor	Rice	Flood	[307,308]
Anaerobic germination	*OsTPP7* encoding Trehalose-6-phosphate Phosphatase	Rice	Flood	[309]
Internode elongation under submergence	*SK1* (*SNORKEL1*), *SK2* encoding, *AP2/ERF* family transcription factor	Rice	Flood	[294]
Internode elongation under submergence	*SD1* (*OsGA20ox2*) encoding, Gibberellin 20-oxidase	Rice	Flood	[310]
Leaf hydrophobicity and gas films are conferred by a wax synthesis gene (*LGF1*); formation of gas films necessary for gas exchange and underwater photosynthesis	Leaf Gas Film 1 (*LGF1*)	Rice	Flood	[311]
Traits: Dormancy/quiescence during submergence; reduced elongation growth and carbohydrate consumption during submergence	*SUB1*	Rice	Flood	[308]
Underwater photosynthesis: Leaf gas films to facilitate gas exchange; supply of carbohydrates to roots for survival, regeneration, and growth	*LGF1/OsHSD1*	Rice	Flood	[311,312]
Chlorophyll retention under submergence: Blocking ethylene responsiveness; scavenging reactive oxygen species (ROS) to protect chlorophyll and other cellular membranes	*SUB1*	Rice	Flood	[313]
Chlorophyll retention under submergence: Scavenging reactive oxygen species (ROS) to protect chlorophyll and other cellular membranes	Several scavengers induced during submergence	Rice	Flood	[313,314]
A barrier to radial oxygen loss (ROL): Minimize oxygen losses in the basal portion of the roots and maximize its delivery to the root apex; minimize uptake of toxins generated in anoxic soils		Rice	Flood	[315,316]
Ion Homeostasis: The excess salt is either transported to the vacuole or sequestered in older tissues which eventually are sacrificed, thereby protecting the plant from salinity stress	SOS1, SOS2, and SOS3 proteins involved in Salt Overly Sensitive (SOS) signaling pathway; SOS1-regulating Na^+^ efflux at the cellular level. It also facilitates long-distance transport of Na^+^ from root to shoot. SOS2 encodes a serine/threonine kinase, and is activated by salt stress elicited Ca^+^ signals. SOS3 is a myristoylated Ca^+^ binding protein	Wheat	Salinity	[317]
	HKT (histidine kinase transporter) located on the plasma membrane and intracellular/tonoplast-localized NHX- encoding K^+^ transporters	Rice	Salinity	[318,319]
Polyamines (PA)*: Protect cells from stress-induced damages, membrane integrity, regulation of gene expression for the synthesis of osmotically active solutes, reduction in ROS production, and controlling the accumulation of Na^+^ and Cl^−^ ions in different organs. * PA is a small, low molecular weight, ubiquitous, polycationic aliphatic molecule that is widely distributed throughout the plant kingdom.	Diamine putrescine (PUT), triamine spermidine (SPD), and tetra-amine spermine (SPM)	Wheat, barley rice	Salinity	[320,321]
Nitric Oxide: Triggers expression of many redox-regulated genes, preventing lipid oxidation, scavenging superoxide radicals, and formation of peroxynitrite that can be neutralized by other cellular processes; activation of antioxidant enzymes	Sodium nitroprusside (SNP), a NO donor	Maize	Salinity	[322]
Hormone Regulation	ABA: The accumulation of ABA can mitigate the inhibitory effect of salinity on photosynthesis, growth, and translocation of assimilates; ABA is involved in the expression of several salt and water deficit-responsive genes including *HVP1* and *HVP10* genes, *TIP 1* and *GLP 1* genes, NCP1 and ZmPIF3 proteins	Rice Wheat Barley Maize	Salinity and drought	[205,323,324,325,326]
	Compounds that have hormonal properties such as salicylic acid (SA), jasmonates, and brassinosteroids (BR)	Rice Wheat Legumes	Salinity and drought	[327,328,329]

## Data Availability

The study did not report any data.

## Supplementary Materials

The following are available online at https://www.mdpi.com/article/10.3390/ijms222312970/s1.

## References

[1] Bailey-Serres J., Parker J.E., Ainsworth E.A., Oldroyd G.E.D., Schroeder J.I. (2019). Genetic strategies for improving crop yields. Nature.

[2] Mickelbart M.V., Hasegawa P.M., Bailey-Serres J. (2015). Genetic mechanisms of abiotic stress tolerance that translate to crop yield stability. Nat. Rev. Genet..

[3] Sita K., Sehgal A., HanumanthaRao B., Nair R.M., Prasad P.V.V., Kumar S., Gaur P.M., Farooq M., Siddique K.H.M., Varshney R.K. (2017). Food legumes and rising temperatures: Effects, adaptive functional mechanisms specific to reproductive growth stage and strategies to improve heat tolerance. Front. Plant Sci..

[4] Priya M., Siddique K.H.M., Dhankher O.P., Prasad P.V.V., Humnath Rao B., Nair R.M., Nayyar H. (2018). Molecular breeding approaches involving physiological and reproductive traits for heat tolerance in food crops. Indian J. Plant Physiol..

[5] Kumar J., Sen Gupta D., Djalovic I., Kumar S., Siddique K.H.M. (2020). Root-omics for drought tolerance in cool-season grain legumes. Physiol. Plant..

[6] Rani A., Devi P., Jha U.C., Sharma K.D., Siddique K.H.M., Nayyar H. (2020). Developing climate-resilient chickpea involving physiological and molecular approaches with a focus on temperature and drought stresses. In Front. Plant Sci..

[7] Singh A.K., Kumar M., Choudhary D., Aher L., Rane J., Singh N.P., Gosal S.S., Wani S.H. (2018). Virus induced gene silencing approach: A potential functional genomics tool for rapid validation of function of genes associated with abiotic stress tolerance. Biotechnologies of Crop Improvement, Volume 2: Transgenic Approaches.

[8] Singh A.K., Kumar M., Choudhary D., Aher L., Rane J., Singh N.P., Gosal S.S., Wani S.H. (2018). RNAi approach: A powerful technique for gene function studies and enhancing abiotic stress tolerance in crop plants. Biotechnologies of Crop Improvement, Volume 2: Transgenic Approaches.

[9] Mousavi-Derazmahalleh M., Bayer P.E., Hane J.K., Valliyodan B., Nguyen H.T., Nelson M.N., Erskine W., Varshney R.K., Papa R., Edwards D. (2019). Adapting legume crops to climate change using genomic approaches. Plant Cell Environ..

[10] Meena K.K., Sorty A.M., Bitla U.M., Choudhary K., Gupta P., Pareek A., Singh D.P., Prabha R., Sahu P.K., Gupta V.K. (2017). Abiotic stress responses and microbe-mediated mitigation in plants: The omics strategies. Front. Plant Sci..

[11] Govindasamy V., George P., Kumar M., Aher L., Raina S.K., Rane J., Annapurna K., Minhas P.S. (2020). Multi-trait PGP rhizobacterial endophytes alleviate drought stress in a senescent genotype of sorghum [*Sorghum bicolor* (L.) Moench]. 3 Biotech.

[12] Minhas P.S., Rane J., Kumar R. (2017). Abiotic Stress Management for Resilient Agriculture.

[13] Lamers J., Meer T., van der Testerink C. (2020). How plants sense and respond to stressful environments. Plant Physiol..

[14] Fahad S., Bajwa A.A., Nazir U., Anjum S.A., Farooq A., Zohaib A., Sadia S., Nasim W., Adkins S., Saud S. (2017). Crop Production under Drought and Heat Stress: Plant Responses and Management Options. Front Plant Sci.

[15] Nayyar H., Sehgal A., Sharma K.S., Siddique K.H.M., Kumar R., Bhogireddy S., Varshney R.K., HanumanthaRao B., Nair R.M., Prasad P.V.V. (2018). Drought or/and heat-stress effects on seed filling in food crops: Impacts on functional biochemistry, seed yield and nutritional quality. Front. Plant Sci..

[16] Arif Y., Singh P., Siddiqui H., Bajguz A., Hayat S. (2020). Salinity induced physiological and biochemical changes in plants: An omic approach towards salt stress tolerance. Plant Physiol. Biochem..

[17] Satpute G.K., Ratnaparkhe M.B., Chandra S., Kamble V.G., Kavishwar R., Singh A.K., Gupta S., Devdas R., Arya M., Singh M., Giri B., Sharma M.P. (2020). Breeding and Molecular Approaches for Evolving Drought-Tolerant Soybeans. Plant Stress Biology: Strategies and Trends.

[18] Rane J., Singh A.K., George P., Govindasamy V., Cukkemane A., Raina S.K., Chavan M.P., Aher L., Sunoj V.J., Singh N.P. (2019). Effect of cow urine-based bioformulations on growth and physiological responses in mungbean under soil moisture stress conditions. Proc. Natl. Acad. Sci. India.

[19] Rane J., Raina S.K., Govindasamy V., Bindumadhava H., Hanjagi P., Giri R., Jangid K.K., Kumar M., Nair R.M. (2021). Use of phenomics for differentiation of mungbean (*Vigna radiata* L. Wilczek) genotypes varying in growth rates per unit of water. Front Plant Sci..

[20] Singh A.K., George P., Aher L., Kumar M., Rane J., Sareen S., Sharma P., Singh C., Jasrotia P., Singh G.P., Sarial A.K. (2021). Genomics, molecular breeding, and phenomics approaches for improvement of abiotic stress tolerance in wheat. Improving Cereal Productivity through Climate Smart Practices.

[21] Scholes R.J. (2020). The Future of Semi-Arid Regions: A Weak Fabric Unravels. Climate.

[22] Grašič M., Golob A., Vogel-Mikuš K., Gaberščik A. (2019). Severe Water Deficiency during the Mid-Vegetative and Reproductive Phase Has Little Effect on Proso Millet Performance. Water.

[23] Prasad P.V.V., Pisipati S.R., Mutava R.N., Tuinstra M.R. (2008). Sensitivity of grain sorghum to high temperature stress during reproductive development. Crop Sci..

[24] Estrada-Campuzano G., Miralles D.J., Slafer G.A. (2008). Genotypic variability and response to water stress of pre- and post-anthesis phases in triticale. Eur. J. Agron..

[25] Plaut Z., Butow B., Blumenthal C., Wrigley C. (2004). Transport of dry matter into developing wheat kernels and its contribution to grain yield under post-anthesis water deficit and elevated temperature. Field Crop Res..

[26] Nadeem M., Li J., Yahya M., Sher A., Ma C., Wang X., Qiu L. (2019). Research Progress and Perspective on Drought Stress in Legumes: A Review. Int. J. Mol. Sci..

[27] Araus J.L., Slafer G.A., Reynolda M.P., Royo C. (2002). Plant breeding and drought in C3 cereals: What should we breed for?. Ann. Bot..

[28] Daryanto S., Wang L., Jacinthe P.A. (2016). Global synthesis of drought effects on maize and wheat production. PLoS ONE.

[29] Daryanto S., Wang L., Jacinthe P.A. (2017). Global synthesis of drought effects on cereal, legume, tuber and root crops production: A review. Agric. Water Manag..

[30] Anwaar H.A., Perveen R., Mansha M.Z., Abid M., Sarwar Z.M., Aatif H.M., ud din Umar U., Sajid M., Aslam H.M.U., Alam M.M. (2020). Assessment of grain yield indices in response to drought stress in wheat (*Triticum aestivum* L.). Saudi J. Biol. Sci..

[31] Zhang J., Zhang S., Cheng M., Jiang H., Zhang X., Peng C., Lu X., Zhang M., Jin J. (2018). Effect of drought on agronomic traits of rice and wheat: A meta-analysis. Int. J. Environ. Res. Public Health.

[32] Carrijo D.R., Lundy M.E., Linquist B.A. (2017). Rice yields and water use under alternate wetting and drying irrigation: A meta-analysis. Field Crop. Res..

[33] Yang X., Wang B., Chen L., Li P., Cao C. (2019). The different influences of drought stress at the flowering stage on rice physiological traits, grain yield, and quality. Sci. Rep..

[34] Swamy B.P.M., Shamsudin N.A.A., Rahman S.N.A., Mauleon R., Ratnam W., StaCruz M.T., Kumar A. (2017). Association mapping of yield and yield-related traits under reproductive stage drought stress in rice (*Oryza sativa* L.). Rice.

[35] Sah R.P., Chakraborty M., Prasad K., Pandit M., Tudu V.K., Chakravarty V.K., Narayan S.C., Rana M., Moharana D. (2020). Impact of water deficit stress in maize: Phenology and yield components. Sci Rep..

[36] Hussain H.A., Men S., Hussain S., Chen Y., Ali S., Zhang S., Zhang K., Li Y., Xu Q., Liao C. (2019). Interactive effects of drought and heat stresses on morpho-physiological attributes, yield, nutrient uptake and oxidative status in maize hybrids. Sci Rep..

[37] Mi N., Cai F., Zhang Y., Ji R., Zhang S., Wang Y. (2018). Differential responses of maize yield to drought at vegetative and reproductive stages. Plant Soil Environ..

[38] Samarah N.H. (2005). Effects of drought stress on growth and yield of barley. Agron. Sustain. Develop..

[39] Alghabari F., Ihsan M. (2018). Effects of drought stress on growth, grain filling duration, yield and quality attributes of barley (*Hordeum vulgare* L.). Bangladesh J. Bot..

[40] Debieu M., Sine B., Passot S., Grondin A., Akata E., Gangashetty P., Vadez V., Gantet P., Foncéka D., Cournac L. (2018). Response to Early Drought Stress and Identification of QTLs Controlling Biomass Production under Drought in Pearl Millet. PLoS ONE.

[41] Halilou O., Assefa Y., Falalou H., Abdou H., Archirou B.F., Karami S.M.A., Jagadish S.V.K. (2020). Agronomic performance of pearl millet genotypes under variable phosphorus, water, and environmental regimes. Agrosyst. Geosci. Environ..

[42] Mafakheri A., Siosemardeh A., Bahramnejad B., Struik P.C., Sohrabi Y. (2010). Effect of drought stress on yield, proline and chlorophyll contents in three chickpea cultivars. Aust. J. Crop Sci..

[43] Pang J., Turner N.C., Du Y.L., Colmer T.D., Siddique K.H.M. (2017). Pattern of water use and seed yield under terminal drought in chickpea genotypes. Front. Plant Sci..

[44] Jan M., Haq T., Sattar H., Butt M., Khaliq A., Arif M., Rauf A. (2020). Evaluation and screening of promising drought tolerant chickpea (*Cicer arietinum* L.) genotypes based on physiological and biochemical attributes under drought conditions. Pak. J. Agricult. Res..

[45] Smith M.R., Veneklaas E., Polania J., Rao I.M., Beebe S.E., Merchant A. (2019). Field drought conditions impact yield but not nutritional quality of the seed in common bean (*Phaseolus vulgaris* L.). PLoS ONE.

[46] Vanaja M., Maheswari M., Sathish P., Vagheera P., Jyothi Lakshmi N., Vijay Kumar G., Yadav S.K., Razzaq A., Singh J., Sarkar B. (2015). Genotypic variability in physiological, biomass and yield response to drought stress in pigeonpea. Physiol. Mol. Biol. Plants.

[47] Basal O., Szabo A. (2020). Physiology, yield and quality of soybean as affected by drought stress. Asian J. Agric. Biol..

[48] Wright J., Hicks D., Naeve S. Predicting the Last Irrigation for Corn and Soybeans in Central Minnesota. https://blog-crop-news.extension.umn.edu/2018/08/predicting-last-irrigation-for-corn-and.html.

[49] Zare M., Ghahremaninejad M., Bazrafshan F. (2012). Influence of drought stress on some traits in five mung bean (*Vigna radiata* (L.) R. Wilczek) genotypes. Int. J. Agron. Plant Prod..

[50] Wang B., Liu C., Zhang D., He C., Zhang J., Li Z. (2019). Effects of maize organ-specific drought stress response on yields from transcriptome analysis. BMC Plant Biol..

[51] Çakir R. (2004). Effect of water stress at different development stages on vegetative and reproductive growth of corn. Field Crop. Res..

[52] Mutava R.N., Prasad P.V.V., Tuinstra M.R., Kofoid K.D., Yu J. (2011). Characterization of sorghum genotypes for traits related to drought tolerance. Field Crops Res..

[53] Mukami A., Ngetich A., Mweu C., Oduor R.O., Muthangya M., Mbinda W.M. (2019). Differential characterization of physiological and biochemical responses during drought stress in finger millet varieties. Physiol. Mol. Biol. Plants.

[54] Gunes A., Inal A., Adak M.S., Bagci E.G., Cicek N., Eraslan F. (2008). Effect of drought stress implemented at pre- or post-anthesis stage on some physiological parameters as screening criteria in chickpea cultivars. Russ. J. Plant Physiol..

[55] Gurumurthy S., Sarkar B., Vanaja M., Lakshmi J., Yadav S.K., Maheswari M. (2019). Morpho-physiological and biochemical changes in black gram (*Vigna mungo* L. Hepper) genotypes under drought stress at flowering stage. Acta Physiol. Plant..

[56] Demirtaş Ç., Yazgan S., Candogan B.N., Sincik M., Büyükcangaz H., Göksoy A.T. (2010). Quality and yield response of soybean (*Glycine max* L. Merrill) to drought stress in sub-humid environment. Afr. J. Biotechnol..

[57] Porter J.R., Semenov M.A. (2005). Crop responses to climatic variation. Phil. Trans. Royal Soc. London. Ser. B Biol. Sci..

[58] Hasanuzzaman M., Nahar K., Alam M.M., Roychowdhury R., Fujita M. (2013). Physiological, biochemical, and molecular mechanisms of heat stress tolerance in plants. Int. J. Mol. Sci..

[59] Prasad P.V.V., Bheemanahalli R., Jagadish S.V.K. (2017). Field crops and the fear of heat stress-Opportunities, challenges and future directions. Field Crop Res..

[60] Hatfield J.L., Prueger J.H. (2015). Temperature extremes: Effect on plant growth and development. Weather. Clim. Extrem..

[61] Djanaguiraman M., Narayanan S., Erdayani E., Prasad P.V.V. (2020). Effects of high temperature stress during anthesis and grain filling periods on photosynthesis, lipids and grain yield in wheat. BMC Plant Biol..

[62] Mäkinen H., Kaseva J., Trnka M., Balek J., Kersebaum K.C., Nendel C., Gobin A., Olesen J.E., Bindi M., Ferrise R. (2018). Sensitivity of European Wheat to Extreme Weather. Field Crop. Res..

[63] Impa S.M., Raju B., Hein N.T., Sandhu J., Prasad P.V.V., Walia H., Jagadish S.V.K. (2021). High Night Temperature Effects on Wheat and Rice: Current Status and Way Forward. Plant Cell Environ..

[64] Semenov M.A., Martre P., Jamieson P.D. (2009). Quantifying effects of simple wheat traits on yield in water-limited environments using a modelling approach. Agric. For. Meteorol..

[65] Spiertz J.H.J., Hamer R.J., Xu H., Primo-Martin C., Don C., van der Putten P.E.L. (2006). Heat stress in wheat (*Triticum aestivum* L.): Effects on grain growth and quality traits. Eur. J. Agron..

[66] Blum A., Kuvela N., Nguyen H.T. (2001). Wheat cellular thermotolerance is related to yield under heat stress. Euphytica.

[67] Salvucci M.E., Crafts-Brandner S.J. (2004). Relationship between the heat tolerance of photosynthesis and the thermal stability of Rubisco activase in plants from contrasting thermal environments. Plant Physiol..

[68] Prasad P.V.V., Boote K.J., Allen L.H., Sheehy J.E., Thomas J.M.G. (2006). Species, ecotype and cultivar differences in spikelet fertility and harvest index of rice in response to high temperature stress. Field Crop Res..

[69] Jagadish S.V.K., Raveendran M., Oane R., Wheeler T.R., Heuer S., Bennett J., Craufurd P.Q. (2010). Physiological and proteomic approaches to address heat tolerance during anthesis in rice (*Oryza sativa* L.). J. Exp. Bot..

[70] Mamrutha H.M., Rinki K., Venkatesh K., Gopalareddy K., Khan H., Mishra C.N., Kumar S., Kumar Y., Singh G., Singh G.P. (2020). Impact of high night temperature stress on different growth stages of wheat. Plant Physiol. Rep..

[71] Prasad P.V.V., Djanaguiraman M. (2014). Response of floret fertility and individual grain weight of wheat to high temperature stress: Sensitive stages and thresholds for temperature and duration. Funct. Plant Biol..

[72] Singh V., Nguyen C.T., van Oosterom E.J., Chapman S.C., Jordan D.R., Hammer G.L. (2015). Sorghum genotypes differ in high temperature responses for seed set. Field Crop. Res..

[73] Opole R.A., Prasad P.V.V., Djanaguiraman M., Vimala K., Kirkham M.B., Upadhyaya H.D. (2018). Thresholds, sensitive stages and genetic variability of finger millet to high temperature stress. J. Agron. Crop Sci..

[74] Pathak H., Kumar M., Molla K.A., Chakraborty K. (2021). Abiotic Stresses in Rice Production: Impacts and Management. Oryza.

[75] Talukder S.K., Babar A.M., Vijayalakshmi K., Poland J., Prasad P.V.V., Bowden R., Fritz A.K. (2014). Mapping QTLs for the traits associated with heat tolerance in wheat (*Triticum aestivum* L.). BMC Genet..

[76] Pradhan G.P., Prasad P.V.V. (2015). Evaluation of wheat chromosome translocation lines for high temperature stress tolerance at grain filling stage. PLoS ONE.

[77] Djanaguiraman M., Perumal R., Jagadish S.V.K., Ciampitti I.A., Welti R., Prasad P.V.V. (2018). Sensitivity of sorghum pollen and pistil to high temperature stress. Plant Cell Environ..

[78] Sunoj V.S.J., Somayananda I.M., Chiluwal A., Perumal R., Prasad P.V.V., Jagadish S.V.K. (2017). Resilience of pollen and post-flowering response in diverse sorghum genotypes exposed to heat stress under field conditions. Crop Sci..

[79] Djanaguiraman M., Perumal R., Ciampitti I.A., Gupta S.K., Prasad P.V.V. (2018). Quantifying pearl millet response to high temperature stress: Thresholds, sensitive stages, genetic variability and relative sensitivity of pollen and pistil. Plant Cell Environ..

[80] Devasirvatham V., Gaur P.M., Mallikarjuna N., Raju T.N., Trethowan R.M., Tan D.K.Y. (2013). Reproductive biology of chickpea response to heat stress in the field is associated with the performance in controlled environments. Field Crop Res..

[81] Awasthi R., Gaur P., Turner N.V., Vadez V., Siddique K.H.M., Nayyar H. (2017). Effects of individual and combined heat and drought stress during seed filling on the oxidative metabolism and yield of chickpea (*Cicer arietinum*) genotypes differing in heat and drought tolerance. Crop Pasture Sci..

[82] Anitha Y., Vanaja M., Kumar G.V. (2015). Identification of attributes contributing to high temperature tolerance in blackgram (*Vigna mungo* L. Hepper) genotypes. Int. J. Sci. Res..

[83] Kaur R., Bains T.S., Rao H.B., Nayyar H. (2015). Responses of mungbean (*Vigna radiata* L.) genotypes to heat stress: Effects on reproductive biology, leaf function and yield traits. Sci. Horticul..

[84] Prasad P.V.V., Boote K.J., Allen L.H., Thomas J.M.G. (2002). Effects of elevated temperature and carbon dioxide on seed-set and yield of kidney bean (*Phaseolus vulgaris* L.). Glob. Chang. Biol..

[85] Djanaguiraman M., Schapaugh W.T., Fritschi F.B., Nguyen H.T., Prasad P.V.V. (2019). Reproductive success of soybean (*Glycine max* L. Merril) cultivars and exotic lines under high daytime temperature. Plant Cell Environ..

[86] Prasad P.V.V., Craufurd P.Q., Summerfield R.J. (1999). Fruit number in relation to pollen production and viability in groundnut exposed to short episodes of heat stress. Ann. Bot..

[87] Prasad P.V.V., Craufurd P.Q., Summerfield R.J., Wheeler T.R. (2020). Effects of short episodes of heat stress on flower production and fruit-set of groundnut (*Arachis hypogaea* L.). J. Exp. Bot.

[88] Prasad P.V.V., Craufurd P.Q., Kakani V.G., Wheeler T.R., Boote K.J. (2001). Influence of high temperature during pre- and post-anthesis stages of floral development on fruit-set and pollen germination in peanut. Aust. J. Plant Physiol..

[89] Prasad P.V.V., Boote K.J., Allen L.H., Thomas J.M.G. (2003). Super-optimal temperatures are detrimental to peanut (*Arachis hypogaea* L.) reproductive processes and yield at both ambient and elevated carbon dioxide. Glob. Chang. Biol..

[90] Bhandari K., Sita K., Sehgal A., Bhardwak A., Gaur P., Kumar S., Singh S., Siddique K.H., Prasad P.V.V., Nayyar H. (2020). Differential heat sensitivity of two cool-season legumes, chickpea and lentil, at reproductive stage, is associated with response in pollen function, photosynthetic ability and oxidative damage. J. Agron. Crop Sci..

[91] Bhardwaj A., Sita K., Sehgal A., Bhandari K., Kumar S., Prasad P.V.V., Jha U., Kumar J., Siddique K.H.M., Nayyar H. (2021). Heat priming of lentil (*Lens culinaris* Medik.) seeds and foliar treatment with γ-aminobutyric acid (GABA), confers protection to reproductive function and yield under high-temperature stress environments. Int. J. Mol. Sci..

[92] Akman Z. (2009). Comparison of high temperature tolerance in maize, rice and sorghum seeds, by plant growth regulators. J. Anim. Vet. Adv..

[93] Narayanan S., Prasad P.V.V., Welti R. (2016). Wheat leaf lipids during heat stress: II. Lipid experiencing coordinated metabolism are detected by analysis of lipid co-occurrence. Plant Cell Environ..

[94] Narayanan S., Tamura P., Roth M., Prasad P.V.V., Welti R. (2016). Wheat leaf lipids during heat stress: I. high day and night temperatures result in major lipid alternations. Plant Cell Environ..

[95] Djanaguiraman M., Boyel D., Welti R., Jagadish S.V.K., Prasad P.V.V. (2018). Decreased photosynthetic rate under high temperature in wheat is due to lipid saturation, oxidation, acylation, and damage to cell organelles. BMC Plant Biol..

[96] Sun A., Somayananda I., Sunoj V.S.J., Singh K., Prasad P.V.V., Gill K., Jagadish S.V.K. (2018). Heat stress during flowering affects time of day of flowering, seed-set and grain quality in spring wheat (*Triticum aestivum* L.). Crop Sci..

[97] Suwa R., Hakata H., Hara H., El-Shemy H.A., Adu-Gyamfi J.J., Nguyen N.T., Kanai S., Lightfoot D.A., Mohapatra P.K., Fujita K. (2010). High temperature effects on photosynthate partitioning and sugar metabolism during ear expansion in maize (*Zea mays* L.) genotypes. Plant Physiol. Biochem..

[98] Prasad P.V.V., Djanaguiraman M. (2011). High night temperature decreases leaf photosynthesis and pollen function in grain sorghum. Funct. Plant Biol..

[99] Prasad P.V.V., Djanaguiraman M., Perumal R., Ciampitti I.A. (2015). Impact of high temperature stress on floret fertility and individual grain weight of grain sorghum: Sensitive stages and thresholds for temperature and duration. Front. Plant Sci..

[100] Djanaguiraman M., Prasad P.V.V., Murugan M., Perumal R., Reddy U.K. (2014). Physiological differences among sorghum (*Sorghum bicolor* L. Moench) genotypes under high temperature stress. Environ. Exp. Bot..

[101] Djanaguiraman M., Nair R., Giraldo J.P., Prasad P.V.V. (2018). Cerium oxide nanoparticles decrease drought-induced oxidative damage in sorghum leading to higher photosynthesis and grain yield. ACS Omega.

[102] Djanaguiraman M., Belliraj N., Bossmann S., Prasad P.V.V. (2018). High temperature stress alleviation by selenium nanoparticles treatment in grain sorghum. ACS Omega.

[103] Devasirvatham V., Gaur P.M., Mallikarjuna N., Tokachichu R.N., Trethowan R.M., Tan D.K.Y. (2012). Effect of high temperature on the reproductive development of chickpea genotypes under controlled environments. Funct. Plant Biol..

[104] Soltani A., Weraduwage S.M., Sharkey T.D., Lowry D.B. (2019). Elevated temperatures cause loss of seed set in common bean (*Phaseolus vulgaris* L.) potentially through the disruption of source-sink relationships. BMC Genom..

[105] Djanaguiraman M., Prasad P.V.V., Al-Khatib K. (2011). Ethylene perception inhibitor 1-MCP decreases oxidative damage of leaves through enhanced antioxidant defense mechanisms in soybean plants grown under high temperature stress. Exp. Environ. Bot..

[106] Djanaguiraman M., Prasad P.V.V., Schapaugh W.T. (2013). High day and night temperature alters leaf assimilation, reproductive success and phosphatidic acid of pollen grain in soybean (*Glycine max* L. Merr.). Crop Sci..

[107] Fahad S., Hussain S., Saud S., Hassan S., Ihsan Z., Shah A.N., Wu C., Yousaf M., Nasim W., Alharby H. (2016). Exogenously applied plant growth regulators enhance the morpho-physiological growth and yield of rice under high temperature. Front. Plant Sci..

[108] Posch B.C., Kariyawasam B.C., Bramley H., Coast O., Richards R.A., Reynolds M.P., Trethowan R., Atkin O.K. (2019). Exploring high temperature responses of photosynthesis and respiration to improve heat tolerance in wheat. J. Exp. Bot..

[109] Rotundo J.L., Tang T., Messina C.D. (2019). Response of maize photosynthesis to high temperature: Implications for modeling the impact of global warming. Plant Physiol. Biochem..

[110] Chavez-Arias C.C., Ligarreto-Moreno G.A., Restrepo-Díaz H. (2018). Evaluation of heat stress period duration and the interaction of daytime temperature and cultivar on common bean. Environ. Exp. Bot..

[111] Zhou R., Yu X., Huang S., Song X., Rosenqvist E., Ottosen C.O. (2020). Genotype-dependent responses of chickpea to high temperature and moderately increased light. Plant Physiol. Biochem..

[112] Guoju X., Fengju Z., Juying H., Chengke L., Jing W., Fei M., Yubi Y., Runyuan W., Zhengji Q. (2016). Response of bean cultures’ water use efficiency against climate warming in semiarid regions of China. Agric. Water Manag..

[113] Bita C.E., Gerats T. (2013). Plant tolerance to high temperature in a changing environment: Scientific fundamentals and production of heat stress-tolerant crops. Front. Plant Sci..

[114] Jagadish S.V., Craufurd P.Q., Wheeler T.R. (2007). High temperature stress and spikelet fertility in rice (*Oryza sativa* L.). J. Exp. Bot..

[115] Bheemanahalli R., Sunoj V.S., Saripalli G., Prasad P.V.V., Balyan H.S., Gupta P.K., Grant V., Gill K.S., Jagadish S.V.K. (2019). Quantifying the impact of heat stress on pollen germination, seed-set and grain filling in spring wheat. Crop Sci..

[116] Ashraf M.A., Akbar A., Parveen A., Rasheed R., Hussain I., Iqbal M. (2018). Phenological application of selenium differentially improves growth, oxidative defense and ion homeostasis in maize under salinity stress. Plant Physiol. Biochem..

[117] Zaki R.N., Radwan T.E.E. (2019). Improving wheat grain yield and its quality under salinity conditions at a newly reclaimed soil by using different organic sources as soil or foliar applications. J. Appl. Sci. Res..

[118] Marcos M., Sharifi H., Grattan S.R., Linquist B.A. (2018). Spatio-temporal salinity dynamics and yield response of rice in water-seeded rice fields. Agric. Water Manag..

[119] Oyiga B.C., Sharma R.C., Shen J., Baum M., Ogbonnaya F.C., Léon J., Ballvora A. (2016). Identification and characterization of salt tolerance of wheat germplasm using a multivariable screening approach. J. Agron. Crop Sci..

[120] Xu S., Hu B., He Z., Ma F., Feng J., Shen W., Yang J. (2011). Enhancement of salinity tolerance during rice seed germination by presoaking with hemoglobin. Int. J. Mol. Sci..

[121] Akbarimoghaddam H., Galavi M., Ghanbari A., Panjehkeh N. (2011). Salinity effects on seed germi- nation and seedling growth of bread wheat cultivars. Trakia J. Sci..

[122] Khodarahmpour Z. (2012). Evaluation of drought stress effects on germination and early growth of inbred lines of MO17 and B. Afr. J. Microbiol. Res..

[123] Yang F., Chen H., Liu C., Li L., Liu L., Han X., Wan Z., Sha A. (2020). Transcriptome profile analysis of two *Vicia faba* cultivars with contrasting salinity tolerance during seed germination. Sci. Rep..

[124] Lavrenko S.O., Lavrenko N.M., Lykhovyd P.V. (2019). Effect of degree of salinity on seed germination and initial growth of chickpea (*Cicer arietinum*). Biosyst. Divers..

[125] Ji J., Shi S., Chen W., Xie T., Du C., Sun J., Shi Z., Gao R., Jiang Z., Xiao W. (2020). Effects of exogenous γ-Aminobutyric acid on the regulation of respiration and protein expression in germinating seeds of mungbean (*Vigna radiata*) under salt conditions. Electron. J. Biotechnol..

[126] Breria C.M., Hsieh C.-H., Yen T.-B., Yen J.-Y., Noble T.J., Schafleitner R. (2020). A SNP-based genome-wide association study to mine genetic loci associated to salinity tolerance in mungbean (*Vigna radiata* L.). Genes.

[127] James R.A., Blake C., Byrt C.S., Munns R. (2011). Major genes for Na+ exclusion, Nax1 and Nax2 (wheat HKT1;4 and HKT1;5), decrease Na+ accumulation in bread wheat leaves under saline and waterlogged conditions. J. Exp. Bot..

[128] Grattan S.R., Grieve C.M. (1998). Salinity-mineral nutrient relations in horticultural crops. Sci. Hortic..

[129] Fahad S., Bano A. (2012). Effect of salicylic acid on physiological and biochemical characterization of maize grown in saline area. Pak. J. Bot..

[130] Flagella Z., Trono D., Pompa M., Di Fonzo N., Pastore D. (2006). Seawater stress applied at germination affects mitochondrial function in durum wheat (*Triticum durum*) early seedlings. Funct. Plant Biol..

[131] Munns R., Tester M. (2008). Mechanisms of salinity tolerance. Annu. Rev. Plant Biol..

[132] Hussain S., Bai Z., Huang J., Cao X., Zhu L., Zhu C., Khaskheli M.A., Zhong C., Jin Q., Zhang J. (2019). 1-methylcyclopropene modulates physiological, biochemical, and antioxidant responses of rice to different salt stress levels. Front. Plant Sci..

[133] de la Reguera E., Veatch J., Gedan K., Tully K.L. (2020). The effects of saltwater intrusion on germination success of standard and alternative crops. Environ. Exp. Bot..

[134] Sadji-Ait Kaci H., Chaker-Haddadj A., Aid F. (2018). Enhancing of symbiotic efficiency and salinity tolerance of chickpea by phosphorus supply. Acta Agric. Scand. Sect. B Soil Plant Sci..

[135] Krishnamurthy S.L., Sharma P.C., Sharma S.K., Batra V., Kumar V., Rao L.V.S. (2016). Effect of salinity and use of stress indices of morphological and physiological traits at the seedling stage in rice. Indian J. Exp. Biol..

[136] Munns R., Rawson H. (1999). Effect of salinity on salt accumulation and reproductive development in the apical meristem of wheat and barley. Funct. Plant Biol..

[137] Forieri I., Hildebrandt U., Rostas M. (2015). Salinity stress effects on direct and indirect defence metabolites in maize. Environ. Exp Bot..

[138] Mukami A., Ngetich A., Syombua E.D., Oduor R.O. (2020). Varietal differences in physiological and biochemical responses to salinity stress in six finger millet plants. Physiol. Mol. Biol. Plants.

[139] Singla R., Garg N. (2005). Influence of salinity on growth and yield attributes in chickpea cultivars. Turkish. J. Agri. For..

[140] Ahmed R., Azeem M., Ahmed N. (2015). Seed germination and seedling growth of pigeon pea (*Cajanus cajan* (L.) Millspaugh) at different salinity regims. Int. J. Biol. Biotech..

[141] Win K.T., Oo A.Z. (2017). Salt-stress-induced changes in protein profiles in two blackgram (*Vigna mungo* L.) varieties differing salinity tolerance. Adv. Plants Agric. Res..

[142] Sehrawat N., Yadav M., Bhat K.V., Sairam R.K., Jaiwal P. (2015). K Effect of salinity stress on mungbean [*Vigna radiata* (L.) Wilczek] during consecutive summer and spring seasons. J. Agric. Sci..

[143] Semida W.M., Rady M.M. (2014). Presoaking application of propolis and maize grain extracts alleviates salinity stress in common bean (*Phaseolus vulgaris* L.). Sci. Hortic..

[144] Patz J.A., Campbell-Lendrum D., Holloway T., Foley J.A. (2005). Impact of regional climate change on human health. Nature.

[145] Herzog M., Striker G.G., Colmer T.D., Pedersen O. (2016). Mechanisms of waterlogging tolerance in wheat—A review of root and shoot physiology. Plant Cell Environ..

[146] Fukao T., Barrera-Figueroa B.E., Juntawong P., Peña-Castro J.M. (2019). Submergence and Waterlogging Stress in Plants: A Review Highlighting Research Opportunities and Understudied Aspects. Front. Plant Sci..

[147] Mustroph A. (2018). Improving Flooding Tolerance of Crop Plants. Agronomy.

[148] Zheng C., Jiang D., Liu F., Dai T., Jing Q., Cao W. (2009). Effects of salt and waterlogging stresses and their combination on leaf photosynthesis, chloroplast ATP synthesis, and antioxidant capacity in wheat. Plant Sci..

[149] Zandalinas S.I., Mittler R., Balfagón D., Arbona V., Gómez-Cadenas A. (2018). Plant adaptations to the combination of drought and high temperatures. Physiol. Plant..

[150] Mittler R. (2006). Abiotic stress, the field environment and stress combination. Trends Plant Sci..

[151] Giraud E., Ho L.H.M., Clifton R., Carroll A., Estavillo G., Tan Y.F., Howell K.A., Ivanova A., Pogson B.J., Millar A.H. (2008). The absence of alternative oxidase1a in *Arabidopsis* results in acute sensitivity to combined light and drought stress. Plant Physiol..

[152] Haghjou M.M., Shariati M., Smirnoff N. (2009). The effect of acute high light and low temperature stresses on the ascorbate-glutathione cycle and superoxide dismutase activity in two *Dunaliella salina* strains. Physiol. Plant..

[153] Prasad P.V.V., Pisipati S.R., Momčilović I., Ristic Z. (2011). Independent and combined effects of high temperature and drought stress during grain filling on plant yield and chloroplast EF-Tu expression in spring wheat. J. Agron. Crop Sci..

[154] Pradhan G.P., Prasad P.V.V., Fritz A.K., Kirkham M.B., Gill B.S. (2012). Effects of drought and high temperature stress on synthetic hexaploid wheat. Funct. Plant Biol..

[155] Pérez-López U., Miranda-Apodaca J., Muñoz-Rueda A., Mena-Petite A. (2013). Lettuce production and antioxidant capacity are differentially modified by salt stress and light intensity under ambient and elevated CO. J. Plant Physiol..

[156] Iyer N.J., Tang Y., Mahalingam R. (2013). Physiological, biochemical and molecular responses to a combination of drought and ozone in *Medicago truncatula*. Plant Cell Environ..

[157] Mahrookashani A., Siebert S., Hüging H., Ewert F. (2017). Independent and combined effects of high temperature and drought stress around anthesis on wheat. J. Agron. Crop Sci..

[158] Rane J., Pannu R.K., Sohu V.S., Saini R.S., Mishra S., Shoran¸ J., Crossa K., Vargas M., Joshi A.K. (2007). Performance of yield and stability of advanced wheat genotypes under heat stress environments of the Indo-Gangetic Plains. Crop Sci..

[159] Chen J., Xu Y., Fei K., Wang R., He J., Fu L., Shao S., Li K., Zhu K., Zhang W. (2020). Physiological mechanism underlying the effect of high temperature during anthesis on spikelet-opening of photo-thermo-sensitive genic male sterile rice lines. Sci Rep..

[160] Prasad P.V.V., Boote K.J., Allen L.H., Thomas J.M.G. (2006). Adverse high temperature effects on pollen viability, seed-set, seed yield and harvest index of grain sorghum (*Sorghum bicolor* L.) are more severe at elevated carbon dioxide due to higher tissue temperatures. Agric. Forest Meteorol..

[161] Bhandari K., Siddique K.H., Turner N.C., Kaur J., Singh S., Agrawal S.K., Nayyar H. (2016). Heat stress at reproductive stage disrupts leaf carbohydrate metabolism, impairs reproductive function, and severely reduces seed yield in lentil. J. Crop Improv..

[162] Genc Y., Taylor J., Lyons G., Li Y., Cheong J., Appelbee M., Oldach K., Sutton T. (2019). Bread wheat with high salinity and sodicity tolerance. Front. Plant Sci..

[163] Ponnambalam P., Manzoor Q., Ragab R., Awadis A., Abdul M.G., Khalaf A. (2016). Tolerance of faba bean, chickpea and lentil to salinity: Accessions’ salinity response functions. Irrig. Drain..

[164] Dramalis C., Katsantonis D., Koutroubas S.D. (2021). Rice growth, assimilate translocation, and grain quality in response to salinity under Mediterranean conditions. AIMS Agric. Food.

[165] Thitisaksakul M., Tananuwong K., Shoemaker C.F., Chun A., Tanadul O.U.M., Labavitch J.M., Beckles D.M. (2015). Effects of timing and severity of salinity stress on rice (*Oryza sativa* L.) yield, grain composition, and starch functionality. J. Agric. Food Chem..

[166] Ploschuk R.A., Miralles D.J., Colmer T.D., Ploschuk E.L., Striker G.G. (2018). Waterlogging of winter crops at early and late stages: Impacts on leaf physiology, growth and yield. Front. Plant Sci..

[167] Arduini I., Baldanzi M., Pampana S. (2019). Reduced growth and nitrogen uptake during waterlogging at tillering permanently affect yield components in late sown oats. Front. Plant Sci..

[168] Farkas Z., Varga-Laszlo E., Anda A., Veisz O., Varga B. (2020). Effects of waterlogging, drought and their combination on yield and water-use efficiency of five Hungarian winter wheat varieties. Water.

[169] Tian L., Li J., Bi W., Zuo S., Li L., Li W., Sun L. (2019). Effects of waterlogging stress at different growth stages on the photosynthetic characteristics and grain yield of spring maize (*Zea mays* L.) under field conditions. Agric. Water Manag..

[170] Sedaghatmehr M., Thirumalaikumar V.P., Kamranfar I., Marmagne A., Masclaux-Daubresse C., Balazadeh S. (2019). A regulatory role of autophagy for resetting the memory of heat stress in plants. Plant Cell Environ..

[171] Zandalinas S.I., Fichman Y., Devireddy A.R., Sengupta S., Azad R.K., Mittler R. (2020). Systemic signaling during abiotic stress combination in plants. Proc. Nat. Acad. Sci. USA.

[172] Richards R.A. (2006). Physiological traits used in the breeding of new cultivars for water-scarce environments. Agric. Water Manag..

[173] Levitt J. (1980). Response of plants to environmental stresses: Chilling, freezing, and high temperature stresses. Physiol. Ecol. Ser. Monogr. Texts Treatises.

[174] Levitt J. (1980). Responses of Plants to Environmental Stresses (Physiological Ecology): Chilling, Freezing, and High Temperature Stresses.

[175] Terletskaya N.V., Lee T.E., Altayeva N.A., Kudrina N.O., Blavachinskaya I.V., Erezhetova U. (2020). Some mechanisms modulating the root growth of various wheat species under ssmotic-stress conditions. Plants.

[176] Roonprapant P., Arunyanark A., Chutteang C. (2021). Morphological and Physiological Responses to Water Deficit Stress Conditions of Robusta Coffee (Coffea Canephora) Genotypes in Thailand. Agric. Nat. Resour..

[177] Ouyang W., Yin X., Yang J., Struik P.C. (2020). Comparisons with wheat reveal root anatomical and histochemical constraints of rice under water-deficit stress. Plant Soil.

[178] Gomes F.P., Oliva M.A., Mielke M.S., de Almeida A.A.F., Leite H.G., Aquino L.A. (2008). Photosynthetic limitations in leaves of young Brazilian Green Dwarf coconut (*Cocos nucifera* L. ‘nana’) palm underwell-watered conditions or recovering from drought stress. Environ. Exp. Bot..

[179] Muktadir M.A., Adhikari K.N., Merchant A., Belachew K.Y., Vandenberg A., Stoddard F.L., Khazaei H. (2020). Physiological and biochemical basis of faba bean breeding for drought adaptation—A review. Agronomy.

[180] Iqbal A., Fahad S., Iqbal M., Alamzeb M., Ahmad A., Anwar S., Khan A.A., Arif M., Saeed M., Song M., Hasanuzzaman M., Tanveer M. (2020). Special Adaptive Features of Plant Species in Response to Drought. Salt and Drought Stress Tolerance in Plants.

[181] Comas L.H., Becker S.R., Cruz V.M.V., Byrne P.F., Dierig D.A. (2013). Root traits contributing to plant productivity under drought. Front Plant Sci..

[182] Polania J., Rao I.M., Cajiao C., Grajales M., Rivera M., Velasquez F., Raatz B., Beebe S.E. (2017). Shoot and root traits contribute to drought resistance in recombinant inbred lines of MD 23–24 × SEA 5 of common bean. Front. Plant Sci..

[183] Orosa-Puente B., Leftley N., von Wangenheim D., Banda J., Srivastava A.K., Hill K., Truskina J., Bhosale R., Morris E., Srivastava M. (2018). Root branching toward water involves posttranslational modification of transcription factor ARF. Science.

[184] Fromm H. (2019). Root plasticity in the pursuit of water. Plants.

[185] Abdelraheem A., Esmaeili N., O’Connell M., Zhang J. (2019). Progress and perspective on drought and salt stress tolerance in cotton. Ind. Crop Prod..

[186] Zhang X., Mi Y., Mao H., Liu S., Chen L., Qin F. (2020). Genetic variation in ZmTIP1 contributes to root hair elongation and drought tolerance in maize. Plant Biotechnol. J..

[187] Uga Y., Sugimoto K., Ogawa S., Rane J., Ishitani M., Hara H., Kitomi Y., Inukai Y., Ono K., Kanno N. (2013). Control of root system architecture by DEEPER ROOTING 1 increases rice yield under drought conditions. Nat. Genet..

[188] Han J., Xiaofen Y., Chang J., Yang G.X. (2017). Overview of the Wheat Genetic Transformation and Breeding Status in China. Methods Mol. Biol..

[189] Sattar A., Sher A., Ijaz M., Ul-Allah S., Rizwan M.S., Hussain M., Jabran K., Cheema M.A. (2020). Terminal drought and heat stress alter physiological and biochemical attributes in flag leaf of bread wheat. PLoS ONE.

[190] Golldack D., Li C., Mohan H., Probst N. (2014). Tolerance to drought and salt stress in plants: Unraveling the signaling networks. Front Plant Sci..

[191] Gangola M.P., Ramadoss B.R. (2018). Sugars play a critical role in abiotic stress tolerance in plants. Biochemical, Physiological and Molecular Avenues for Combating Abiotic Stress in Plants.

[192] Sami F., Yusuf M., Faizan M., Faraz A., Hayat S. (2016). Role of sugars under abiotic stress. Plant Physiol. Biochem..

[193] Signorelli S. (2016). The Fermentation Analogy: A Point of View for Understanding the Intriguing Role of Proline Accumulation in Stressed Plants. Front. Plant Sci..

[194] Signorelli S., Tarkowski Ł.P., Van den Ende W., Bassham D.C. (2019). Linking autophagy to abiotic and biotic stress responses. Trends Plant Sci..

[195] Xiaochuang C., Chu Z., Lianfeng Z., Junhua Z., Hussain S., Lianghuan W., Qianyu J. (2017). Glycine increases cold tolerance in rice via the regulation of N uptake, physiological characteristics, and photosynthesis. Plant Physiol. Biochem..

[196] Choudhury F.K., Rivero R.M., Blumwald E., Mittler R. (2017). Reactive oxygen species, abiotic stress and stress combination. Plant J..

[197] Huang H., Ullah F., Zhou D.X., Yi M., Zhao Y. (2019). Mechanisms of ROS regulation of plant development and stress responses. Front. Plant Sci..

[198] Nadarajah K.K. (2020). Ros homeostasis in abiotic stress tolerance in plants. Int. J. Mol. Sci..

[199] Corpas F.J., del Río L.A., Palma J.M. (2019). Plant peroxisomes at the crossroad of NO and H_2_O_2_ metabolism. J. Integr. Plant Biol..

[200] Rezayian M., Ebrahimzadeh H., Niknam V. (2020). Nitric oxide stimulates antioxidant system and osmotic adjustment in soybean under drought stress. J. Soil Sci. Plant Nutr..

[201] Biswas M.S., Fukaki H., Mori I.C., Nakahara K., Mano J. (2019). Reactive oxygen species and reactive carbonyl species constitute a feed-forward loop in auxin signaling for lateral root formation. Plant J..

[202] Parvin K., Nahar K., Hasanuzzaman M., Bhuyan M.H.M.B., Mohsin S.M., Fujita M. (2020). Exogenous vanillic acid enhances salt tolerance of tomato: Insight into plant antioxidant defense and glyoxalase systems. Plant Physiol. Biochem..

[203] Chung Y.S., Kim K.-S., Hamayun M., Kim Y. (2020). Silicon confers soybean resistance to salinity stress through regulation of reactive oxygen and reactive nitrogen species. Front. Plant Sci..

[204] Djanaguiraman M., Prasad P.V.V., Seppanen M. (2010). Selenium protects sorghum leaves from oxidative damage under high temperature stress by enhancing antioxidant defense system. Plant Physiol. Biochem..

[205] Bousba R., Rached-Kanouni M., Benghersallah N., Djekoune A., Ykhlef N. (2020). Role of exogenous application of abscisic acid ABA in drought tolerance and evaluation of antioxidant activity in durum wheat genotypes. Acta Sci. Nat..

[206] Haq S.U., Kumari D., Dhingra P., Kothari S.L., Kachhwaha S. (2020). Variant biochemical responses: Intrinsic and adaptive system for ecologically different rice varieties. J. Crop Sci. Biotechnol..

[207] Yin N., Zhang Z., Wang L., Qian K. (2016). Variations in organic carbon, aggregation, and enzyme activities of gangue-fly ash-reconstructed soils with sludge and arbuscular mycorrhizal fungi during 6-year reclamation. Envi. Sci. Pollut. Res..

[208] Broad R.C., Bonneau J.P., Hellens R.P., Johnson A.A.T. (2020). Manipulation of ascorbate biosynthetic, recycling, and regulatory pathways for improved abiotic stress tolerance in plants. Internat. J. Mol. Sci..

[209] Dumanović J., Nepovimova E., Natić M., Kuča K., Jaćević V. (2021). The Significance of Reactive Oxygen Species and Antioxidant Defense System in Plants: A Concise Overview. Front. Plant Sci..

[210] Hasanuzzaman M., Alhaithloul H.A.S., Parvin K., Bhuyan M.H.M.B., Tanveer M., Mohsin S.M., Nahar K., Soliman M.H., Al Mahmud J., Fujita M. (2019). Polyamine action under metal/metalloid stress: Regulation of biosynthesis, metabolism, and molecular interactions. Int. J. Mol. Sci..

[211] Almeselmani M., Deshmukh P.S., Sairam R.K., Kushwaha S.R., Singh T.P. (2006). Protective role of antioxidant enzymes under high temperature stress. Plant Sci..

[212] Ullah N., Ababaei B., Chenu K. (2020). Increasing Heat Tolerance in Wheat to Counteract Recent and Projected Increases in Heat Stress. Proceedings.

[213] Sharma S., Singh A., Singh B. (2019). Characterization of in vitro antioxidant activity, bioactive components, and nutrient digestibility in pigeon pea (*Cajanus cajan*) as influenced by germination time and temperature.. J. Food Biochem.

[214] Singh D.P., Singh V., Gupta V.K., Shukla R., Prabha R., Sarma B.K., Patel J.S. (2020). Microbial inoculation in rice regulates antioxidative reactions and defense related genes to mitigate drought stress. Sci. Rep..

[215] Yang Z., Wang Y., Wei X., Zhao X., Wang B., Sui N. (2017). Transcription Profiles of Genes Related to Hormonal Regulations Under Salt Stress in Sweet Sorghum. Plant Mol. Biol. Report..

[216] Liu Q., Liang Z., Feng D., Jiang S., Wang Y., Du Z., Hu G., Zhang P., Ma Y., Lohmann J.U. (2021). Transcriptional landscape of rice roots at the single-cell resolution. Mol. Plant..

[217] Thameur A., Ferchichi A., López-Carbonell M. (2011). Quantification of free and conjugated abscisic acid in five genotypes of barley (*Hordeum vulgare* L.) under water stress conditions. South Afr. J. Bot..

[218] Hamayun M., Khan S.A., Shinwari Z.K., Khan A.L., Ahmad N., Lee I.J. (2010). Effect of Polyethylene Glycol Induced Drought Stress on Physio-Hormonal Attributes of Soybean. Pak. J. Bot..

[219] Guóth A., Tari I., Gallé Á., Csiszár J., Pécsváradi A., Cseuz L., Erdei L. (2009). Comparison of the drought stress responses of tolerant and sensitive wheat cultivars during grain filling: Changes in flag leaf photosynthetic activity, ABA levels, and grain yield. J. Plant Growth Regul..

[220] Yu J., Cang J., Lu Q., Fan B., Xu Q., Li W., Wang X. (2020). ABA enhanced cold tolerance of wheat ‘dn1′ via increasing ROS scavenging system. Plant Signal Behav..

[221] Bheemanahalli R., Sathishraj R., Manoharan M., Sumanth H.N., Muthurajan R., Ishimaru T., Jagadish SVK (2017). Is early morning flowering an effective trait to minimize heat stress damage during flowering in rice?. Field Crop Res..

[222] Bheemanahalli R., Impa S.M., Krassovskaya I., Vennapusa A.R., Gill K.S., Obata T., Jagadish S.V.K. (2020). Enhanced N-metabolites, ABA and IAA -conjugate in anthers instigate heat sensitivity in spring wheat. Physiol. Plant..

[223] Elhakem A.H. (2020). Salicylic acid ameliorates salinity tolerance in maize by regulation of phytohormones and osmolytes. Plant Soil Environ..

[224] Gharsallah C., Fakhfakh H., Grubb D., Gorsane F. (2016). Effect of salt stress on ion concentration, proline content, antioxidant enzyme activities and gene expression in tomato cultivars. AoB Plants.

[225] Guajardo E., Correa J.A., Contreras-Porcia L. (2016). Role of abscisic acid (ABA) in activating antioxidant tolerance responses to desiccation stress in intertidal seaweed species. Planta.

[226] Rhaman M.S., Imran S., Rauf F., Khatun M., Baskin C.C., Murata Y., Hasanuzzaman M. (2020). Seed priming with phytohormones: An effective approach for the mitigation of abiotic stress. Plants.

[227] Wei L., Wang L., Yang Y., Wang P., Guo T., Kang G. (2015). Abscisic acid enhances tolerance of wheat seedlings to drought and regulates transcript levels of genes encoding ascorbate-glutathione biosynthesis. Front. Plant Sci..

[228] Awan S.A., Khan I., Rizwan M., Zhang X., Brestic M., Khan A., El-Sheikh M.A., Alyemeni M.N., Ali S., Huang L. (2020). Exogenous abscisic acid and jasmonic acid restrain polyethylene glycol-induced drought by improving the growth and antioxidative enzyme activities in pearl millet. Physiol. Plant..

[229] Fujita Y., Fujita M., Shinozaki K., Yamaguchi-Shinozaki K. (2011). ABA-mediated transcriptional regulation in response to osmotic stress in plants. J. Plant Res..

[230] Sharma E., Sharma R., Borah P., Jain M., Khurana J.P., Pandey G. (2015). Emerging Roles of Auxin in Abiotic Stress Responses. Elucidation of Abiotic Stress Signaling in Plants.

[231] Al-Tabbal J.A., Kafawin O.M., Ayad J.Y. (2006). Influence of water stress and plant growth regulators on yield and development of two durum wheat (*Triticum turgidum* L. var. durum) cultivars. Jordan J. Agric. Sci..

[232] Guo J., Xu W., Yu X., Shen H., Li H., Cheng D., Song J. (2016). Cuticular wax accumulation is associated with drought tolerance in wheat near-isogenic lines. Front. Plant Sci..

[233] Hipol R.M., Dalisay T.U., Ardales E.Y., Lourdes M., Cedo O., Cuevas V.C. (2015). Endophytic yeasts possibly alleviate heavy metal stress in their host *Phragmitesaustralis cav*. (Trin.) ex Steud. through the production of plant growth promoting hormones. Bullten. Environ. Pharmacol. Life Sci..

[234] Peleg Z., Blumwald E. (2011). Hormone balance and abiotic stress tolerance in crop plants. Curr. Opin. Plant Biol..

[235] Werner T., Nehnevajova E., Köllmer I., Novák O., Stmad M., Krämer U., Schmülling T. (2010). Root-specific reduction of cytokinin causes enhanced root growth, drought tolerance, and leaf mineral enrichment in Arabidopsis and tobacco. Plant Cell.

[236] Wang J., Song L., Gong X., Xu J., Li M. (2020). Functions of jasmonic acid in plant regulation and response to abiotic stress. Int. J. Mol. Sci..

[237] Reynolds M.P., Balota M., Delgado M.I.B., Amani I., Fischer R.A. (1994). Physiological and Morphological Traits Associated with Spring Wheat Yield Under Hot, Irrigated Conditions. Funct. Plant Biol..

[238] Sadok W., Lopez J.R., Smith K.P. (2021). Transpiration Increases under High-Temperature Stress: Potential Mechanisms, Trade-Offs and Prospects for Crop Resilience in a Warming World. Plant Cell Environ..

[239] Cossani C.M., Reynolds M.P. (2012). Physiological Traits for Improving Heat Tolerance in Wheat. Plant Physiol..

[240] Pinto R.S., Reynolds M.P. (2015). Common Genetic Basis for Canopy Temperature Depression under Heat and Drought Stress Associated with Optimized Root Distribution in Bread Wheat. Theor. Appl. Genet..

[241] Kumar M., Raina S.K., Govindasamy V., Singh A.K., Choudhary R.L., Rane J., Minhas P.S. (2017). Assimilates mobilization, stable canopy temperature and expression of Expansin stabilizes grain weight in wheat cultivar LOK-1 under different soil moisture conditions. Bot. Stud..

[242] Kumar M., Govindasamy V., Rane J., Singh A.K., Choudhary R.L., Raina S.K., George P., Aher L., Singh N.P. (2017). Canopy temperature depression (CTD) and canopy greenness associated with variation in seed yield of soybean genotypes grown in semi-arid environment. South Afr. J. Bot..

[243] Christopher M., Chenu K., Jennings R., Fletcher S., Butler D., Borrell A., Christopher J. (2018). QTL for Stay-Green Traits in Wheat in Well-Watered and Water-Limited Environments. Field Crop. Res.

[244] Dreccer M.F., Fainges J., Whish J., Ogbonnaya F.C., Sadras V.O. (2018). Comparison of sensitive stages of wheat, barley, canola, chickpea and field pea to temperature and water stress across Australia. Agric. For. Meteorol..

[245] Hunt J.R., Hayman P.T., Richards R.A., Passioura J.B. (2018). Opportunities to reduce heat damage in rain-fed wheat crops based on plant breeding and agronomic management. Field Crop. Res..

[246] Crawford A.J., McLachlan D.H., Hetherington A.M., Franklin K.A. (2012). High temperature exposure increases plant cooling capacity. High temperature exposure increases plant cooling capacity. Curr. Biol..

[247] Khan Z., Shahwar D., Roychowdhury R., Choudhury S., Hasanuzzaman M., Srivastava S. (2020). Role of Heat Shock Proteins (HSPs) and Heat Stress Tolerance in Crop Plants. Sustainable Agriculture in the Era of Climate Change.

[248] Lee U., Rioflorido I., Hong S., Larkindale J., Waters E.R., Vierling E. (2007). The *Arabidopsis* ClpB/Hsp100 family of proteins: Chaperones for stress and chloroplast development. Plant J..

[249] Hu W., Hu G., Han B. (2009). Genome-wide survey and expression profiling of heat shock proteins and heat shock factors revealed overlapped and stress specific response under abiotic stresses in rice. Plant Sci..

[250] Huang B., Xu C. (2008). Identification and Characterization of Proteins Associated with Plant Tolerance to Heat Stress. J. Integr. Plant Biol..

[251] Simões-Araújo J.L., Rumjanek N.G., Margis-Pinheiro M. (2003). Small heat shock proteins genes are differentially expressed in distinct varieties of common bean. Braz. J. Plant Physiol..

[252] Danekar P., Tyagi A., Mahto A., Krishna K.G., Singh A., Raje R.S., Gaikwad K., Singh N.K. (2014). Genome wide characterization of hsp 100 family genes from pigeonpea. Indian J. Genet. Plant Breed..

[253] Cui Y., Lu S., Li Z., Cheng J., Hu P., Zhu T., Wang X., Jin M., Wang X., Li L. (2020). Cyclic nucleotide-gated ion channels 14 and 16 promote tolerance to heat and chilling in rice. Plant Physiol..

[254] Mahfouze H.A., Ramadan W.A., Mahfouze S.A. (2019). Gene expression and single nucleotide polymorphisms (SNP) marker of heat shock protein (HSP) gene in wheat (*Triticum aestivum* L.). Plant Arch..

[255] Rodríguez-Vera A.P., Acosta-Gallegos J.A., Ruiz-Nieto J.E., Montero-Tavera V. (2020). Selection by genetic expression profiles of desi and kabuli chickpea (*Cicer arietinum* L.) genotypes tolerant to high temperature stress. Legume Res..

[256] Rasheed R., Wahid A., Farooq M., Hussain I., Basra S.M.A. (2011). Role of proline and glycinebetaine pretreatments in improving heat tolerance of sprouting sugarcane (*Saccharum* sp.) buds. Plant Growth Regul..

[257] Foyer C.H. (2015). Redox homeostasis: Opening up ascorbate transport. Nat. Plants.

[258] Das S., Mohanty S., Dash D., Muduli K.C. (2020). Enhancement of growth and seed yield of rice (Oryza sativa l.) through foliar spray of osmoprotectants under high temperature stress. Ind. J. Tradit. Knowl..

[259] Kumar S., Singh R., Nayyar H. (2013). α-Tocopherol Application Modulates the Response of Wheat (*Triticum aestivum* L.) Seedlings to Elevated Temperatures by Mitigation of Stress Injury and Enhancement of Antioxidants. J. Plant Growth Regul..

[260] Tiwari Y.K., Yadav S.K. (2020). Effect of high-temperature stress on ascorbate–glutathione cycle in maize. Agric. Res..

[261] Venkatesh J., Park S.W. (2014). Role of L-ascorbate in alleviating abiotic stresses in crop plants. Bot. Stud..

[262] Yang C., Fraga H., van Ieperen W., Santos J.A. (2020). Assessing the impacts of recent-past climatic constraints on potential wheat yield and adaptation options under Mediterranean climate in southern Portugal. Agric. Syst..

[263] Wang H.Q., Liu P., Zhang J.W., Zhao B., Ren B.Z. (2020). Endogenous hormones inhibit differentiation of young ears in maize (*Zea mays* L.) under heat stress. Front. Plant Sci..

[264] Harsha A., Sharma Y.K., Joshi U., Rampuria S., Singh G., Kumar S., Sharm R. (2016). Effect of shortterm heat stress on total sugars, proline and some antioxidant enzymes in moth bean (*Vigna aconitifolia*). Ann. Agric. Sci..

[265] Mody T., Bonnot T., Nagel D.H. (2020). Interaction between the circadian clock and regulators of heat stress responses in plants. Genes.

[266] Wu X., Liu L., Xu Q., Wei H., Wang X., Sun W., Zhuge Q. (2020). Characteristics and functions of PePIF3, a gene related to circadian rhythm in “Nanlin 895” poplar. Plant Mol. Biol. Report..

[267] Roy S.J., Negrao S., Tester M. (2014). Salt resistant crop plants. Curr. Opin. Biotechnol..

[268] Hernández J.A. (2019). Salinity tolerance in plants: Trends and perspectives. Int. J. Mol. Sci..

[269] Huang L., Wu D.Z., Zhang G.P. (2020). Advances in studies on ion transporters involved in salt tolerance and breeding crop cultivars with high salt tolerance. J. Zhejiang Univ. Sci. B..

[270] Theerawitaya C., Tisarum R., Samphumphuang T., Takabe T., Cha-um S. (2020). Expression levels of the Na+/K+ transporter OsHKT2;1 and vacuolar Na+/H+ exchanger OsNHX1, Na enrichment, maintaining the photosynthetic abilities and growth performances of indica rice seedlings under salt stress. Physiol. Mol. Biol. Plants.

[271] Zhang M., Liang X., Wang L., Cao Y., Song W., Shi J., Lai J., Jiang C. (2019). A HAK family Na^+^ transporter confers natural variation of salt tolerance in maize. Nat. Plants.

[272] Ami K., Planchais S., Cabassa C., Guivarc’h A., Very A.A., Khelifi M., Djebbar R., Abrous-Belbachir O., Carol P. (2020). Different proline responses of two Algerian durum wheat cultivars to in vitro salt stress. Acta Physiol. Plant..

[273] Chaudhary R., Kumar M., Sengar R.S., Kumar P., Singh S.K., Kumar Y. (2017). Effect of salinity stress on photosynthesis and expression of salt tolerant genes in Chickpea (*Cicer arietinum* L.). Internat. J. Chem. Stud..

[274] Wu H., Shabala L., Azzarello E., Huang Y., Pandolfi C., Su N., Wu Q., Cai S., Bazihizina N., Wang L. (2018). Na + extrusion from the cytosol and tissue-specific Na + sequestration in roots confer differential salt stress tolerance between durum and bread wheat. J. Exp. Bot..

[275] Singh P., Mahajan M.M., Singh N.K., Kumar D., Kumar K. (2020). Physiological and molecular response under salinity stress in bread wheat (*Triticum aestivum* L.). J. Plant Biochem. Biotechnol..

[276] Dietz K.J., Tavakoli N., Kluge C., Mimura T., Sharma S.S., Harris G.C., Chardonnens A.N., Golldack D. (2001). Significance of the V-type ATPase for the adaptation to stressful growth conditions and its regulation on the molecular and biochemical level. J. Exp. Bot..

[277] Wang X., Cai J., Jiang D., Liu F., Dai T., Cao W. (2011). Pre-anthesis high-temperature acclimation alleviates damage to the flag leaf caused by post-anthesis heat stress in wheat. J. Plant Physiol..

[278] Adem G.D., Roy S.J., Huang Y., Chen Z.-H., Wang F., Zhou M., Bowman J.P., Holford P., Shabala S. (2017). Expressing *Arabidopsis thaliana* V-ATPase subunit C in barley (*Hordeum vulgare*) improves plant performance under saline condition by enabling better osmotic adjustment. Funct. Plant Biol..

[279] Gupta A., Shaw B.P. (2020). Biochemical and molecular characterisations of salt tolerance components in rice varieties tolerant and sensitive to NaCl: The relevance of Na+ exclusion in salt tolerance in the species. Functl. Plant Biol..

[280] Carden D., Walker D., Flowers T.J., Miller A.J. (2003). Single cell measurements of the contributions of cytosoloc NA and K to salt tolerance. Plant Physiol..

[281] Rahnama H., Vakilian H., Fahimi H., Ghareyazie B. (2011). Enhanced salt stress tolerance in transgenic potato plants (*Solanum tuberosum* L.) expressing a bacterial mtlD gene. Acta Physiol. Plant..

[282] Jiang Y., Davis A.R., Vujanovic V., Bueckert R.A. (2019). Reproductive development response to high daytime temperature in field pea. J. Agron. Crop Sci..

[283] Kumar V., Wani S.H., Suprasanna P., Tran L.S.P. (2018). Salinity Responses and Tolerance in Plants, Volume 1: Targeting Sensory, Transport and Signaling Mechanisms.

[284] Per T.S., Khan N.A., Reddy P.S., Masood A., Hasanuzzaman M., Khan M.I.R., Anjum N.A. (2017). Approaches in modulating proline metabolism in plants for salt and drought stress tolerance: Phytohormones, mineral nutrients and transgenics. Plant Physiol. Biochem..

[285] Ashraf M., Foolad M.R. (2007). Roles of glycine betaine and proline in improving plant abiotic stress resistance. Env. Exp. Bot..

[286] Munns R., Passioura J.B., Colmer T.D., Byrt C.S. (2020). Osmotic adjustment and energy limitations to plant growth in saline soil. New Phytol..

[287] Jiang C., Cui Q., Feng K., Xu D., Li C., Zheng Q. (2016). Melatonin improves antioxidant capacity and ion homeostasis and enhances salt tolerance in maize seedlings. Acta Physiol. Plant.

[288] Ren J., Ye J., Yin L., Li G., Deng X., Wang S. (2020). Exogenous melatonini salt tolerance by mitigating osmotic, ion, and oxidative stresses in maize seedlings. Agronomy.

[289] Jackson M.B., Ram P.C. (2003). Physiological and molecular basis of susceptibility and tolerance of rice plants to complete submergence. Ann. Bot..

[290] Sairam R.K., Kumutha D., Ezhilmathi K., Dwshmukh P.S. (2008). Physiology and biochemistry of waterlogging tolerance in plants. Biol. Plant.

[291] Kirk G.J.D., Greenway H., Atwell B.J., Ismail A.M., Colmer T.D. (2014). Adaptation of rice to flooded soils. Progr. Bot..

[292] Kaur G., Singh G., Motavalli P.P., Nelson K.A., Orlowski J.M., Golden B.R. (2020). Impacts and management strategies for crop production in waterlogged or flooded soils: A review. Agron. J..

[293] Kuroha T., Ashikari M. (2020). Molecular mechanisms and future improvement of submergence tolerance in rice. Mol. Breed..

[294] Hattori Y., Nagai K., Furukawa S., Song X.-J., Kawano R., Sakakibara H., Wu J., Matsumoto T., Yoshimura A., Kitano H. (2009). The ethylene response factors SNORKEL1 and SNORKEL2 allow rice to adapt to deep water. Nature.

[295] Goyal K., Kaur K., Kaur G. (2020). Foliar treatment of potassium nitrate modulates the fermentative and sucrose metabolizing pathways in contrasting maize genotypes under water logging stress. Physiol. Mol. Biol. Plants.

[296] Boraiah K.M., Basavaraj P.S., Harisha C.B., Kochewad S.A., Khapte P.S., Bhendarkar M.P., Kakade V.D., Rane J., Kulshreshtha N., Pathak H. (2021). Abiotic Stress Tolerant Crop Varieties, Livestock Breeds and Fish Species. No. ICAR-National Institute of Abiotic Stress Management, Baramati, Pune, Maharashtra, India. Tech. Bull..

[297] Luo L., Xia H., Lu B.-R. (2019). Editorial: Crop Breeding for Drought Resistance. Front. Plant Sci..

[298] Shavrukov Y., Kurishbayev A., Jatayev S., Shvidchenko V., Zotova L., Koekemoer F., De Groot S., Soole K., Langridge P. (2017). Early flowering as a drought escape mechanism in plants: How can it aid wheat production?. Front. Plant Sci..

[299] Matyszczak I., Tominska M., Shakhira Z., Christoph D., Hansson M. (2020). Analysis of early-flowering genes at barley chromosome 2H expands the repertoire of mutant alleles at the Mat-c locus. Plant Cell Rep..

[300] Chen G., Hu J., Dong L., Zeng D., Guo L., Zhang G., Zhu L., Qian Q. (2020). The Tolerance of Salinity in Rice Requires the Presence of a Functional Copy of FLN. Biomolecules.

[301] Cui L.H., Byun M.Y., Oh H.G., Kim S.J., Lee J., Park H., Lee H., Kim W.T. (2020). Type II galactinol synthase 2 from antarctic flowering plant *Deschampsia antarctica* and rice improves cold and drought tolerance by accumulation of raffinose family oligosaccharides in transgenic rice plants. Plant Cell Physiol..

[302] Carillo P. (2018). GABA shunt in durum wheat. Front. Plant Sci..

[303] Wang H., Zhou L., Fu Y., Cheung M., Wong F., Phang T., Sun Z., Lam H. (2012). Expression of an apoplast-localized BURP-domain protein from soybean (GmRD22) enhances tolerance towards abiotic stress. Plant Cell Environ..

[304] Gill S.S., Tajrishi M., Madan M., Tuteja N. (2013). A DESD box helicase functions in salinity stress tolerance by improving photosynthesis and antioxidant machinery in rice (*Oryza sativa* L. cv. PB1). Plant Molecular Biol..

[305] Tuteja N., Sahoo R.K., Garg B., Tuteja R. (2013). OsSUV3 dual helicase functions in salinity stress tolerance by maintaining photosynthesis and antioxidant machinery in rice (*Oryza sativa* L. cv. IR64). Plant J..

[306] Begara-Morales J.C., Anchez-Calvo B.S., Chaki M., Valderrama R., Mata-Perez C., Lopez-Jaramillo J., Padilla M.N., Carreras A., Corpas F.J., Barrosa J.B. (2014). Dual regulation of cytosolic ascorbate peroxidase (APX) by tyrosine nitration and S-nitrosylation. J. Expt. Bot..

[307] Bailey-Serres J., Fukao T., Ronald P., Ismail A., Heuer S., Mackill D. (2010). Submergence tolerant rice: SUB1′s journey from landrace to modern cultivar. Rice.

[308] Xu K., Xu X., Fukao T., Canlas P., Maghirang-Rodriguez R., Heuer S., Ismail A.M., Bailey-Serres J., Ronald P.C., Mackill D.J. (2006). Sub1A is an ethylene-responsefactor- like gene that confers submergence tolerance to rice. Nature.

[309] Kretzschmar T., Pelayo M.A., Trijatmiko K.R., Gabunada L.F., Alam R., Jimenez R., Mendioro M.S., Slamet-Loedin I.H., Sreenivasulu N., Bailey-Serres J. (2015). A trehalose-6-phosphate phosphatase enhances anaerobic germination tolerance in rice. Nature.

[310] Kuroha T., Nagai K., Gamuyao R., Wang D.R., Furuta T., Nakamori M., Kitaoka T., Adachi K., Minami A., Mori Y. (2018). Ethylene-gibberellin signaling underlies adaptation of rice to periodic flooding. Science.

[311] Kurokawa Y., Nagai K., Huan P.D., Shimazaki K., Mori Y., Toda Y., Kuroha T., Hayashi N., Aiga S., Itoh J. (2018). Rice leaf hydrophobicity and gas films are conferred by a wax synthesis gene (LGF1) and contribute to flood tolerance. New Phytol..

[312] Pedersen O., Rich S.M., Colmer T.D. (2009). Surviving floods: Leaf gas films improve O(2) and CO(2) exchange, root aeration, and growth of completely submerged rice. Plant J..

[313] Ella E.S., Kawano N., Ito O. (2003). Importance of active oxygen-scavenging system in the recovery of rice seedlings after submergence. Plant Sci..

[314] Dordas C., Rivoal J., Hill R.D. (2003). Plant hemoglobins, nitric oxide and hypoxic stress. Ann Bot..

[315] Colmer T.D., Armstrong W., Greenway H., Ismail A.M., Kirk G.J.D., Atwell B.J. (2014). Physiological mechanisms in flooding tolerance of rice: Transient complete submergence and prolonged standing water. Prog. Bot..

[316] Yamauchi T., Colmer T.D., Pedersen O., Nakazono M. (2018). Regulation of root traits for internal aeration and tolerance to soil waterlogging-flooding stress. Plant Physiol..

[317] Xu C., Wang M., Zhou L., Quan T., Xia G. (2013). Heterologous expression of the wheat aquaporin gene TaTIP2;2 compromises the abiotic stress tolerance of *Arabidopsis thaliana*. PLoS ONE.

[318] Yang C., Zhao N., Xu C., Liu B., Shi D. (2012). Regulation of ion homeostasis in rice subjected to salt and alkali stresses. Aust. J. Crop. Sci..

[319] Gupta B., Huang B. (2014). Mechanism of Salinity Tolerance in Plants: Physiological, Biochemical, and Molecular Characterization. Int. J. Genom..

[320] Darko E., Gierczik K., Hudak O., Forgo P., Pal M., Turkosi E., Kovács V., Dulai S., Majláth I., Molnár I. (2017). Differing metabolic responses to salt stress in wheat-barley addition lines containing different 7H chromosomal fragments. PLoS ONE.

[321] Laha S., Kumar D., Sengupta D.N., Gangopadhyay G. (2019). In silico characterization of SAMdC from Pokkali rice and its overexpression in transgenic tobacco. Vegetos.

[322] Zhang Y.L., Wang Y., Liu Q., Zhang Q., Wei Q., Zhang W. (2006). Nitric oxide enhances salt tolerance in maize seedlings through increasing activities of proton-pump and Na+/H+ antiport in the tonoplast. Planta.

[323] Vishal B., Krishnamurthy P., Rengasamy R., Prakash P.K. (2018). OsTPS8 controls yield-related traits and confers salt stress tolerance in rice by enhancing suberin deposition. New Phytol..

[324] Boussora F., Allam M., Guasmi F., Ferchichi A., Rutten T., Hansson M., Youssef H.M., Borner A. (2019). Spike developmental stages and ABA role in spikelet primordia abortion contribute to the final yield in barley (*Hordeum vulgare* L.). Bot Stud..

[325] Zong N., Wang H., Li Z., Ma L., Xie L., Pang J., Fan Y., Zhao J. (2020). Maize NCP1 negatively regulates drought and ABA responses through interacting with and inhibiting the activity of transcription factor ABP. Plant Mol Biol..

[326] Gao Y., Wu M., Zhang M., Jiang W., Liang E., Zhang D., Zhang C., Xiao N., Chen J. (2018). Roles of a maize phytochrome-interacting factors protein ZmPIF3 in regulation of drought stress responses by controlling stomatal closure in transgenic rice without yield penalty. Plant Mol Biol..

[327] Nadarajah K., Abdul Hamid N.W., Abdul Rahman N.S.N. (2021). SA-Mediated Regulation and Control of Abiotic Stress Tolerance in Rice. Int. J. Mol. Sci..

[328] Sharma M., Gupta S.K., Majumder B., Maurya V.K., Deeba F., Alam A., Pandey V. (2017). Salicylic acid mediated growth, physiological and proteomic responses in two wheat varieties under drought stress. J. Proteomics..

[329] Sirhindi G., Kumar S., Kumar M., Kaur H., Sharma P., Kaur G., Singh V.P., Singh S., Tripathi D.K., Prasad S.M., Bhardwaj R., Chauhan D.K. (2021). Phytohormonal signaling under abiotic stress in legumes. Abiotic Stress and Legumes.

[330] Raina S.K., Singh P.Y., Singh A.K., Raskar N., Rane J., Minhas P.S. (2018). Exogenous gibberellic acid does not induce early flowering in Mungbean [(*Vigna radiata* (L.) Wilczek.]. Legume Res..

[331] Khanna-Chopra R., Singh K., Tripathi B.N., Müller M. (2015). Drought resistance in crops: Physiological and genetic basis of traits for crop productivity. Stress Responses in Plants: Mechanisms of Toxicity and Tolerance.

[332] Zikhali M., Griffiths S., Ogihara Y., Takumi S., Handa H. (2015). The effect of earliness per se (Eps) genes on glowering time in bread wheat. Advances in Wheat Genetics: From Genome to Field.

[333] Hill C.B., Li C. (2016). Genetic architecture of flowering phenology in cereals and opportunities for crop improvement. Front. Plant Sci..

[334] Ibrahim A., Harrison M., Meinke H., Fan Y., Johnson P., Zhou M. (2018). A regulator of early flowering in barley (*Hordeum vulgare* L.). PLoS ONE.

[335] Turner N.C., Wright G.C., Siddique K.H.M. (2001). Adaptation of grain legumes (pulses) to water-limited environments. Adv. Agron..

[336] Serraj R., Bidinger F.R., Chauhan Y.S., Seetharama N., Nigam S.N., Saxena N.P., Kijne J.W., Barker R., Molden D. (2003). Management of drought in ICRISAT cereal and legume mandate crops. Water Productivity in Agriculture: Limits and Opportunities for Improvement.

[337] Van Oosterom E.J., Mahalakshmi V., Bidinger F.R., Rao K.P. (1996). Effect of water availability and temperature on the genotype-by-environment interaction of pearl millet in semi-arid tropical environments. Euphytica.

[338] Zonneveld V.M., Rakha M., Tan S.Y., Chou Y.Y., Chang C.H., Yen J.Y., Schafleitner R., Nair R., Naito K., Solberg S.Ø. (2020). Mapping patterns of abiotic and biotic stress resilience uncovers conservation gaps and breeding potential of *Vigna* wild relatives. Sci. Rep..

[339] Lynch J.P., Strock C.F., Schneider H.M., Sidhu J.S., Ajmera I., Galindo-Castañeda T., Klein S.P., Hanlon M.T. (2021). Root anatomy and soil resource capture. Plant Soil..

[340] Leach K.A., Hejlek L.G., Hearne L.B., Nguyen H.T., Sharp R.E., Davis G.L. (2011). Primary root elongation rate and abscisic acid levels of maize in response to water stress. Crop Sci..

[341] Danakumara T., Kumari J., Singh A.K., Sinha S.K., Pradhan A.K., Sharma S., Jha S.K., Kumar S., Jha G.K., Yadav M.C. (2021). Genetic dissection of seedling root system architectural traits in diverse panel of hexaploid wheat through multi-locus genome-wide association mapping for improving drought tolerance. Int. J. Mol. Sci..

[342] Purushothaman R., Mainassara Z.A., Nalini M., Rajaram P., Krishnamurthy L., Cholenahalli L.G. (2013). Root anatomical traits and their possible contribution to drought tolerance in grain legumes. Plant Prod. Sci..

[343] Phule A.S., Barbadikar K.M., Madhav M.S., Subrahmanyam D., Senguttuvel P., Babu M.B.B.P., Kumar P.A. (2019). Studies on root anatomy, morphology and physiology of rice grown under aerobic and anaerobic conditions. Physiol. Mol. Biol. Plants.

[344] Polania J., Poschenrieder C., Rao I., Beebe S. (2017). Root traits and their potential links to plant ideotypes to improve drought resistance in common bean. Theor. Exp. Plant Physiol..

[345] Rane J., Sharma D., Ekatpure S., Aher L., Kumar M., Prasad S.V., Nankar A.N., Singh N.P. (2019). Relative tolerance of photosystem II in spike, leaf, and stem of bread and durum wheat under desiccation. Photosynthetica.

[346] Das K., Roychoudhury A. (2014). Reactive oxygen species (ROS) and response of antioxidants as ROS-scavengers during environmental stress in plants. Front. Environ. Sci..

[347] Sun A.Z., Guo F.Q. (2016). Chloroplast retrograde regulation of heat stress responses in plants. Front. Plant Sci..

[348] Yoo Y.H., Hong W.J., Jung K.H. (2019). A systematic view exploring the role of chloroplasts in plant abiotic stress responses. Bio. Med. Res. Intern..

[349] Khurana N., Chauhan H., Khurana H. (2015). Characterization of a chloroplast localized wheat membrane protein (TaRCI) and its role in heat, drought and salinity stress tolerance in Arabidopsis thaliana. Plant Gene.

[350] Ashraf A., Rehman O.U., Muzammil S., Léon J., Naz A.A., Rasool F., Ali G.M., Zafar Y., Khan M.R. (2019). Evolution of *Deeper Rooting 1-like* homoeologs in wheat entails the C-terminus mutations as well as gain and loss of auxin response elements. PLoS ONE.

[351] Kitomi Y., Hanzawa E., Kuya N., Inoue H., Hara N., Kawai S., Uga Y. (2020). Root angle modifications by the DRO1 homolog improve rice yields in saline paddy fields. Proc. Nat. Acad. Sci. USA.

[352] Ismail A.M., Singh U.S., Singh S., Dar M., Mackill D.J. (2013). The contribution of submergence-tolerant (Sub1) rice varieties to food security in flood-prone areas. Field Crops Res..

[353] Sarkar R.K., Das K.K., Panda D., Reddy J.N., Patnaik S.S.C., Patra B.C., Singh D.P. (2014). Submergence Tolerance in Rice: Biophysical Constraints, Physiological Basis and Identification of Donors.

[354] Pucciariello C., Perata P. (2013). Quiescence in rice submergence tolerance: An evolutionary hypothesis. Trends Plant Sci..

[355] Niroula R.K., Pucciariello C., Ho V.T., Novi G., Fukao T., Perata P. (2012). SUB1A-dependent and -independent mechanisms are involved in the flooding tolerance of wild rice species. Plant J..

[356] Okishio T., Sasayama D., Hirano T., Akimoto M., Itoh K., Azuma T. (2015). Ethylene is not involved in adaptive responses to flooding in the Amazonian wild rice species *Oryza grandiglumis*. J. Plant Physiol..

[357] Pan Z., Liu M., Zhao H., Tan Z., Liang K., Sun Q., Qiu F. (2020). ZmSRL5 is involved in drought tolerance by maintaining cuticular wax structure in maize. J. Integ. Plant Biol..

[358] Khazaei H., Santanen A., Street K., Stoddard F.L. (2019). Genotypic variation in leaf epicuticular wax quantity in a large faba bean (*Vicia faba* L.) germplasm collection. Plant Genet. Res. Charact. Utiliz..

[359] Jiang H., Feakins S.J., Sun H., Feng X., Zhang X., Liu X. (2020). Dynamic changes in leaf wax n-alkanes and δ13C during leaf development in winter wheat under varied irrigation experiments. Org. Geochem..

[360] Estravis-Barcala M., Mattera M.G., Soliani C., Bellora N., Opgenoorth L., Heer K., Arana M.V. (2020). Molecular basis of responses to abiotic stress in trees. J. Exp. Bot..

[361] Millar A.J., Urquiza U., Freeman P.L., Hume A., Plotkin G.D., Sorokina O., Zardilis A., Zielinski T. (2019). Practical steps to digital organism models, from laboratory model species to “crops in silico”. J. Exp. Bot..

[362] Haak D.C., Fukao T., Grene R., Hua Z., Ivanov R., Perrella G., Li S. (2017). Multi-level regulation of abiotic stress responses in plants. Front. Plant Sci..

[363] Kimotho R.N., Baillo E.H., Zhang Z. (2019). Transcription factors involved in abiotic stress responses in maize (*Zea mays* L) and their roles in enhanced productivity in the post-genomic era. Peer J..

[364] Pandey S. (2020). Plant receptor-like kinase signaling through heterotrimeric G-proteins. J. Exp. Bot..

[365] Tekel S.J., Smith C.L., Lopez B., Mani A., Connot C., Livingstone X., Haynes K.A. (2019). Engineered orthogonal quorum sensing systems for synthetic gene regulation in *Escherichia coli*. Front. Bioeng. Biotechnol..

[366] Gonzalez D.H. (2015). Plant Transcription Factors: Evolutionary, Structural and Functional Aspects. Plant Transcription Factors: Evolutionary, Structural and Functional Aspects.

[367] Hoang X.L.T., Nhi D.N.H., Thu N.B.A., Thao N.P., Tran L.-S.P. (2017). Transcription factors and their roles in signal transduction in plants under abiotic stresses. Curr. Genom..

[368] Oszvald M., Primavesi L.F., Griffiths C.A., Cohn J., Basu S.S., Nuccio M.L., Paul M.J. (2018). Trehalose 6-phosphate regulates photosynthesis and assimilate partitioning in reproductive tissue. Plant Physiol..

[369] Sharan A., Soni P., Nongpiur R.C., Singla-Pareek S.L., Pareek A. (2017). Mapping the ‘two-component system’ network in rice. Sci. Rep..

[370] Lakra N., Kaur C., Anwar K., Singla-Pareek S.L., Pareek A. (2018). Proteomics of contrasting rice genotypes: Identification of potential tar-gets for raising crops for saline environment. Plant Cell Environ..

[371] Gigli-Bisceglia N., Engelsdorf T., Hamann T. (2020). Plant cell wall integrity maintenance in model plants and crop species-relevant cell wall components and underlying guiding principles. Cell Mol. Life Sci..

