# Supplementary figures and images for: The Adaptation and Tolerance of Major Cereals and Legumes to Important Abiotic Stresses
Source: Int J Mol Sci. 2021 Nov 30;22(23):12970. doi: 10.3390/ijms222312970 (PMC8657814; doi:10.3390/ijms222312970)

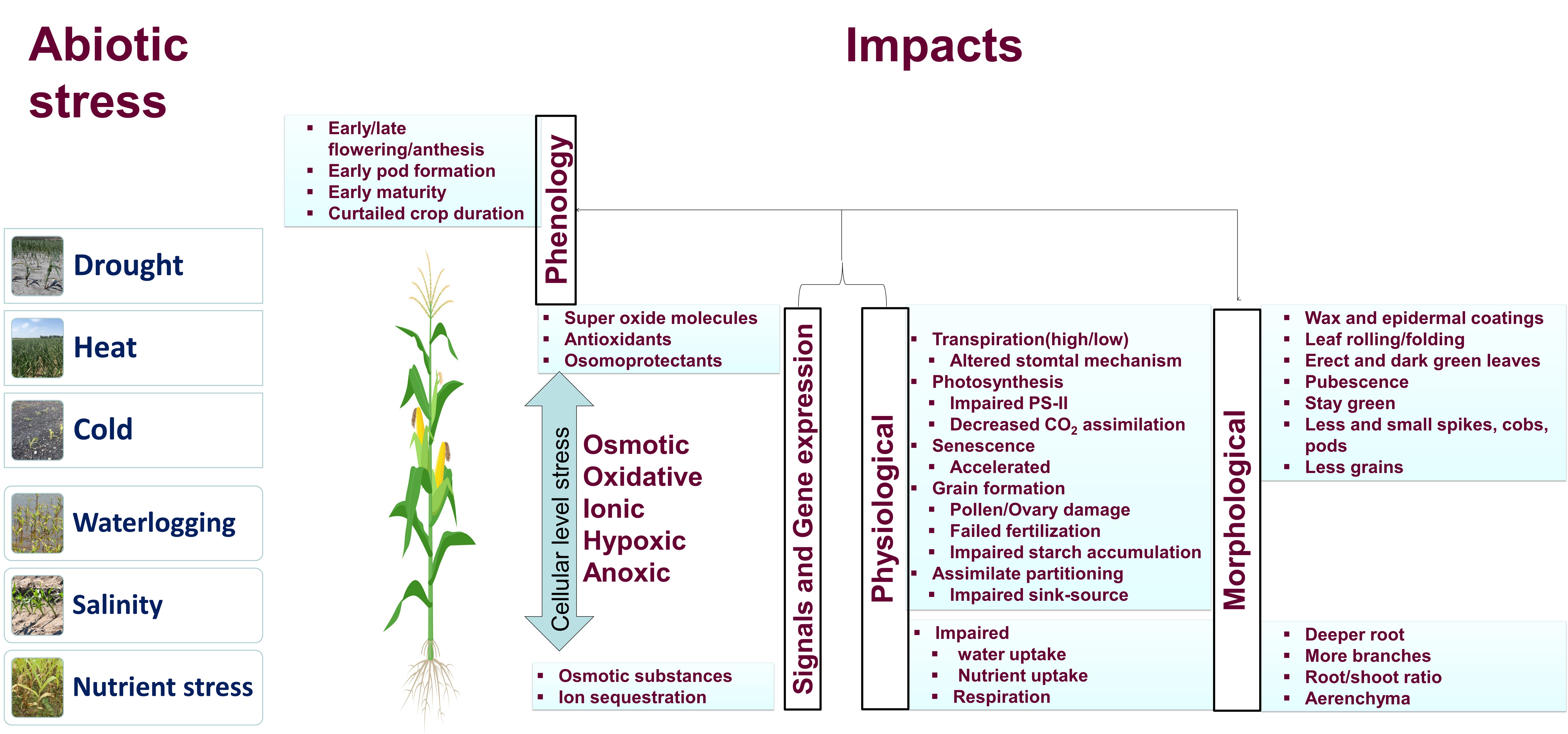

Supplement: Supplementary file 1 [file ijms-22-12970-s001.zip › Fig S1.Abiotic stress and impacts.jpg]

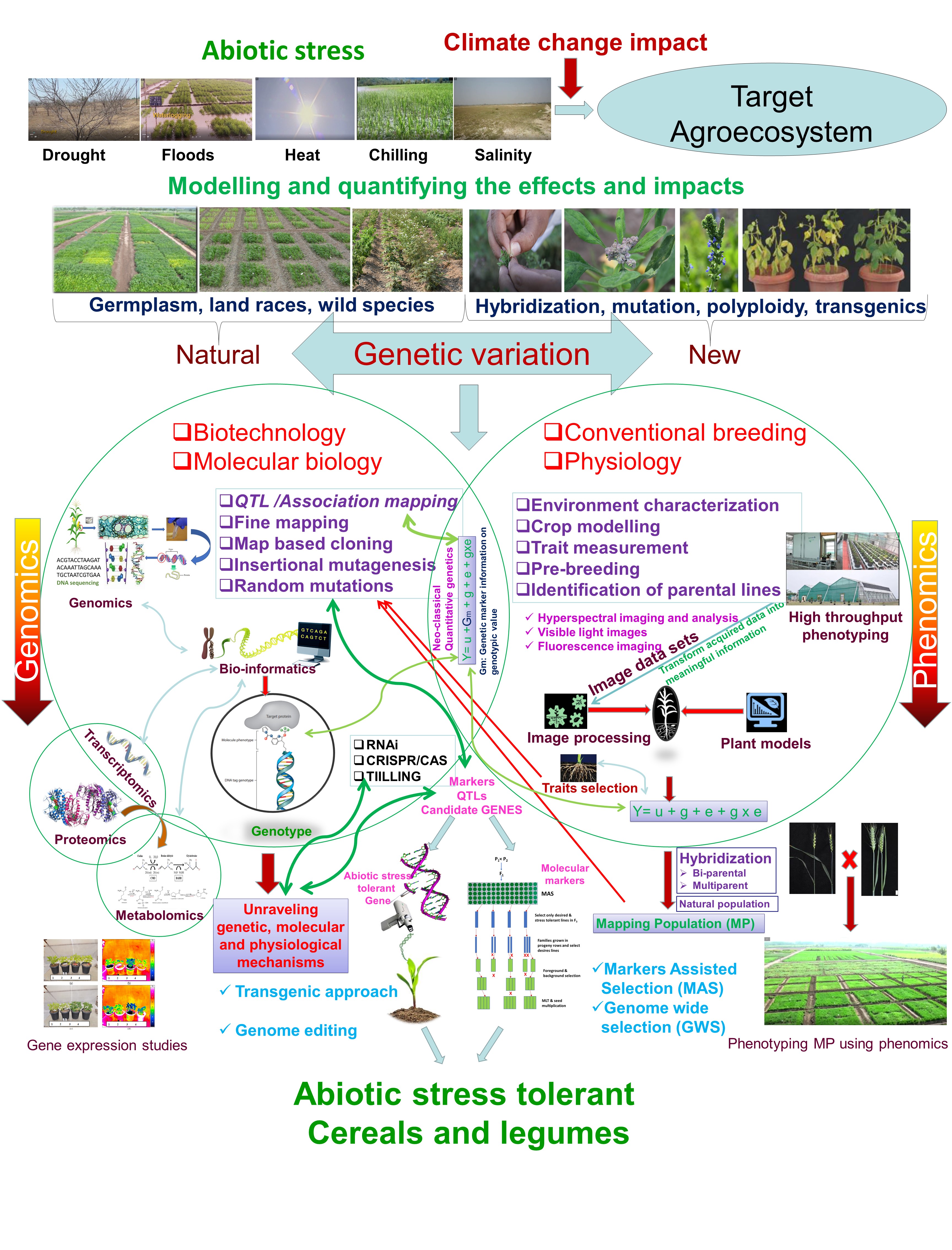

Supplement: Supplementary file 1 [file ijms-22-12970-s001.zip › RevisedFig S2_Model_Strategies.jpg]
